# Host-Defense Peptides Caerin 1.1 and 1.9 Stimulate TNF-Alpha-Dependent Apoptotic Signals in Human Cervical Cancer HeLa Cells

**DOI:** 10.3389/fcell.2020.00676

**Published:** 2020-07-31

**Authors:** Guoying Ni, Shu Chen, Mo Chen, Jialing Wu, Binbin Yang, Jianwei Yuan, Shelley F. Walton, Hejie Li, Ming Q. Wei, Yuejian Wang, Guoqiang Chen, Xiaosong Liu, Tianfang Wang

**Affiliations:** ^1^Cancer Research Institute, First People’s Hospital of Foshan, Foshan, China; ^2^Genecology Research Centre, University of the Sunshine Coast, Maroochydore, QLD, Australia; ^3^The First Affiliated Hospital, School of Clinical Medicine of Guangdong Pharmaceutical University, Guangzhou, China; ^4^Menzies Health Institute Queensland and School of Medical Science, Griffith University, Southport, QLD, Australia; ^5^Department of Laboratory Medicine, Institute of Nanomedicine Technology, Weifang Medical University, Weifang, China; ^6^Department of Mechanical and Biofunctional System, Institute of Industrial Science, The University of Tokyo, Tokyo, Japan

**Keywords:** caerin peptide, HeLa cell, apoptosis, TNF-α signaling pathway, TMT labeling, proteomics, confocal microscopy, western blot

## Abstract

Host defense caerin 1.1 and 1.9 peptides, isolated from the glandular secretion of Australian tree frogs, the genus *Litoria*, have been previously shown to have multiple biological activities, including the inhibition of human papillomavirus (HPV) 16 early protein E7 transformed murine as well as human cancerous cell proliferation both *in vitro* and *in vivo*. However, the mechanism underlying their anti-proliferative activities against HPV18+ cervical cancer HeLa cells remains unknown. This study comparatively investigated the anti-proliferation on HeLa cells by caerin 1.1, 1.9, and their mixture, followed by confocal microscopy examination to assess the cellular intake of the peptides. Tandem mass tag labeling proteomics was employed to reveal the proteins that were significantly regulated by the peptide treatment in cells and cell growth environment, to elucidate the signaling pathways that were modulated. Western blot was performed to confirm the modulation of the pathways. Both caerin 1.1 and 1.9 highly inhibited HeLa cell proliferation with a significant additive effect compared to untreated and control peptide. They entered the cells with different magnitudes. Intensive protein-protein interaction was detected among significantly upregulated proteins. Translation, folding and localization of proteins and RNA processing, apoptosis process was significantly enriched post the treatments. The apoptotic signaling was suggested as a result of tumor necrosis factor-α (TNF-α) pathway activation, indicated by the dose-dependent elevated levels of caspase 3 and caspase 9. The epidermal growth factor receptor and androgen receptor pathways appeared inhibited by the peptides. Moreover, the activation of T-cell receptor derived from the quantitation results further implies the likelihood of recruiting more T cells to the cell growth environment post the treatment and more sensitive to T cell mediated killing of HeLa cells. Our results indicate that caerin 1.1 and 1.9 mediate apoptotic signals of HeLa cells and may subsequently enhances adaptive T cell immune responses.

## Introduction

Cervical cancer is the second most common cancer in woman worldwide, results from persistent infection of Human papillomavirus (HPV) infection, especially HPV subtype 16, 18 infection, which accounts for more than 70% of cervical cancer (Leo et al., 2017; McGregor et al., 2018). HPV prophylactic vaccine introduced a decade ago effectively prevents HPV infection and may eradicate cervical cancer in countries that the vaccines are well distributed (McGregor et al., 2018). However, the vaccine is ineffective for those already infected with HPV and developing countries that have most cervical cancer patients have limited resources to vaccinate those at risk (Ni et al., 2015). Therefore, development of effective cervical cancer therapies remains as an urgent task.

Cancer immunotherapy is becoming a routine cancer treatment option following the introduction of immune checkpoint antibodies (Diesendruck and Benhar, 2017). Therapeutic vaccines target cancer cells through activation or induction of antigen specific T cells, with minimal affection of normal cells and tissues (Ni et al., 2015). The efficacy of therapeutic vaccines is far from satisfactory in clinical trials, up to now, few vaccines such as synthetic long peptide (SLP) vaccines have shown promising results in clinical trials against premalignant lesions, whereas responses against later stage carcinomas have remained inclusive (Galliverti et al., 2018). An ideal therapeutic vaccine should be able to elicit high quality and enough numbers of effective T cells, which can migrate to the tumor site, and overcome the tumor immunosuppressive microenvironment and kill the tumor cells via the process such as apoptosis (Ni et al., 2015).

Tumor necrosis factor alpha (TNF-α) is an inflammatory cytokine associated with both cell survival and apoptosis. The activation of caspases regulates the apoptotic effects of TNFα. Binding of TNF Receptor I stimulates the formation of a cytoplasmic Tumor necrosis factor receptor type 1-associated DEATH domain complex, leading to the activation of caspase 8 and the initiation of an apoptotic signaling cascade (Chau et al., 2004). TNFα-induced apoptosis requires the activation of JNK-dependent pathway (Deng et al., 2003). The modulation of TNF-α pathway to activate apoptosis in cancer cells toward the development of anti-tumor therapy has been widely investigated (Wang et al., 1999; Petersen et al., 2007; Shen et al., 2018).

Our previous study on a murine cell line TC-1 cells, which is a lung cancer cell transformed with HPV16 E6/E7 and commonly used as a surrogate cell line for cervical cancer research in mouse model, has shown that caerin peptides can alter the abundance of several immune-related proteins and related pathways, such as the Tec kinase and ILK signaling pathways, as well as the levels of pro-inflammatory cytokines and chemokines (Ni et al., 2018). Notably, caerin peptides were able to increase the survival time of TC-1 tumor bearing mice after therapeutic vaccination with a HPV16E7 peptide-based vaccine containing IL-10 inhibitor, via recruiting increased amounts of T cells to the tumor site (Pan et al., 2019). However, TC-1 cell is an artificial cell line that cannot represent the cell toxicity result against cervical cancer cells. We have recently found that caerin peptides can inhibit the growth of HeLa cells (Ma et al., 2020) and Siha cells (Data not shown). In this study, the anti-proliferation activity of caerin 1.1 and 1.9 on human HeLa cells was characterized, and the TMT-labeling quantitative proteomic technique was used to compare for variation in protein abundance. The gene ontology enrichment and protein-protein interactions related to significantly regulated proteins were analyzed. In addition, the modulation of biological signaling pathways was elucidated based on protein quantitation and confirmed by western blot analysis.

## Materials and Methods

### Chemicals

Trifluoroacetic acid (TFA), methanol, acetonitrile (ACN), formic acid (FA), NH_4_HCO_3_, urea, dithiothreitol (DTT), iodoacetamide (IAA), RIPA buffer, sodium pyruvate, L-glutamine, G418, lipopolysaccharide and non-essential amino acid solution were obtained from Sigma-Aldrich (St. Louis, MO, United States). Trypsin (V5280) was purchased from Promega (Madison, WI, United States). Ultrapure water was prepared by Milli-Q water purification system (Millipore, Bedford, MA, United States). The TMT6plex^TM^ Mass Tag Isobaric Labeling kit was purchased from Thermo Scientific^TM^ (Madison, WI, United States). Protease inhibitor cocktail was purchased from (GE Healthcare, Little Chalfont, United Kingdom).

### Cell Line, Cell Culture, and Peptide Synthesis

Human HeLa cell line was purchased from Shanghai Institutes for Cell Resource Centre, Chinese Academy of Sciences, and cultured following the protocols in the product sheets. Briefly, HeLa cells were maintained in complete DMEM media (Gibco) supplemented with 10% heat inactivated FBS, 100 U of penicillin/ml and 100 μg of streptomycin/ml and were cultured at 37°C with 5% CO_2_.

Three peptides, free or conjugated with FITC, including caerin 1.1 (GLLSVLGSVAKHVLPHVVPVIAEHL-NH_2_), caerin 1.9 (GLFGVLGSIAKHVLPHVVPVIAEKL-NH_2_) and P3 (GTELPSPPSVWFEAEFK-OH) as a negative control, were synthesized by Mimotopes (Melbourne, Australia). The purity of the peptides was >99% as determined by reverse-phase HPLC.

### MTT Assay and Checkerboard Assay

Cell proliferation was determined by an MTT assay (ATCC, United States) following manufacturer instructions. Briefly, HeLa cells were cultured separately in flat bottomed 96 well plates. Caerin peptides with different concentrations (0, 0.4, 1.9, 3.9, and 5.8 nM), and P3 (0, 0.5, 2.6, 5.2, and 7.8 nM) were added to 1 × 10^4^ of HeLa cells and cultured overnight at 37°C with 5% CO_2_. The wells treated with Triton X-100 (Anitha et al., 2012) and 31 nM caerin 1.1 were used as positive controls. Each treatment was performed in triplicates. Ten microliters of MTT Reagent were added and cultured for another 2 h, then 100 μl of Detergent Reagent was added, and the plate was left at room temperature in the dark for 2 h. Results were analyzed with a microtiter plate reader (BioTek, United States) at 570 nm according to the manufacturer’s protocol. The IC50 value of each caerin peptide was calculated using the method described elsewhere (Ma et al., 2020).

The additive effect of caerin 1.1/1.9 was evaluated using the checkerboard assay (Hsieh et al., 1993; Orhan et al., 2005). It was set up on a 96 well plate, and a series dilution of caerin 1.1 and 1.9 were used to treat 5 × 10^3^ of HeLa cells in combination overnight. For wells along the *x*-axis, caerin 1.9 was diluted (0, 0.95, 1.9, 3.9, 7.8, and 15.6 nM), while caerin 1.1 was diluted along the *y*-axis (0, 0.95, 1.9, 3.9, 7.8, and 15.6 nM). Each well within this 6 × 6 checkerboard contained a combination of caerin 1.1 and 1.9 at different concentrations. The fractional inhibitory concentration index (FICI) for a well was calculated as follows,

$$F\,I\,C\,I=F\,I\,C_{1.1}+F\,I\,C_{1.9}=\frac{A}{I\,C\,50_{1.1}}+\frac{B}{I\,C\,50_{1.9}}$$

Where, *A* is the concentration of caerin 1.1 in a given well along the inhibition-no-inhibition interface; *IC50_1_._1_* is the IC50 of caerin 1.1 alone; *FIC_1_._1_* is the fractional inhibitory concentration of caerin 1.1; and *B*, *IC50_1_._9_*, and *FIC_1_._9_* are defined in the same fashion for caerin 1.9. An *FICI* ≤ 1.0 implies additive effect or synergy.

### Cellular Uptake Assay Using Confocal Microscopy and Identification of Unconjugated Caerin 1.1 and 1.9 in Cytoplasm of Treated HeLa Cells Using uHPLC and Mass Spectral Analysis

The cellular uptake process and capability of caerin 1.1 and 1.9 was evaluated and the FITC-labeled peptides were used for better monitoring of the location and distribution of peptides. HeLa cells were seeded on the 8-well tissue culture chambers (SARSTEDT, Germany) at 5 × 10^4^ cells per well, and then the cells were incubated with culture medium containing 3.9 nM caerin 1.1, 1.9, or 5.2 nM P3 (negative control) each well for 5, 30, and 2 h respectively. After that, HeLa cells were rinsed twice with PBS and stained the nuclei by Hoechst 33342 (blue, Invitrogen) for 15 min, and cell plasma membrane was stained by CellMask (Deep red, Invitrogen) for 15 min. The confocal fluorescence images were acquired with a confocal laser scanning microscopy (CLSM, Olympus FV3000) under ambient conditions. 3D reconstruction and 3D Pearson’s Correlation Coefficient (PCC) calculation were performed using the Imaris 9.2 software (Bitplane AG, Zurich, Switzerland).

To investigate the entry of unconjugated caerin peptides into cytoplasm, HeLa cells were cultured and treated with 10 μg/ml of caerin 1.1 and 1.9. Cells were collected at 1 and 2 h post the treatment and centrifuged at 1,000 × *g*, 4°C for 5 min to separate cells. Cell pellet was washed using 1 mL cold PBS and centrifuged at 1,000 × *g*, 4°C for 5 min, this was repeated twice. The, cell pellet was lysed with RIPA buffer contain protease inhibitor cocktail. The protein extracts were centrifuged at 12,000 × *g* at 4°C for 15 min, and the resulted supernatants were then subjected to ultracentrifugation at 100,000 × *g* at 4°C for 60 min, to remove cell membrane. Since the molecular weights of caerin peptides are below 3 kDa, an ultrafiltration with a cutoff of 10 kDa was used on the supernatants to concentrate the eluates possibly containing these peptides. The eluates were analyzed by using a bioZen Peptide XB-C18 column (2.6 μm, 150 × 2.1 mm) on an Agilent 1290 infinity uHPLC (Agilent, Palo Alto, CA, United States), with the reference to the chromatographs of pure synthetic caerin 1.1 and1.9. In addition, 30 s fraction around the elution time of pure caerin peptides were collected for LC-MS/MS analysis.

### Protein Extraction From Cell Growth Environment

To study the change of protein profiles in cell-growing environment in response to peptide treatment, excretory/secretory proteins (ESPs) were collected and analyzed. Approximately 1 ml cell culture mixture (containing both cells and supernatant) with or without the treatments (the concentration of the peptides was 1.9 nM) was collected and centrifuged at 1,000 × *g*, 4°C for 5 min, to separate cells and supernatant. The supernatant about 0.5 cm above the pellet was collected and centrifuged at 4°C, 12,000 × *g* for 15 min, while the pellet cells were also collected for following proteomic analysis. After the centrifugation, the supernatant was collected, lyophilized and subjected to in-solution digestion as described elsewhere (Ni et al., 2018). Briefly, 500 μg protein in 100 μl lysis buffer (8M urea, 0.8M NH_4_HCO_3_, pH 8.0) were reduced with 100 mM DTT at 37°C, and subsequently alkylated with 100 mM IAA at room temperature (RT) in the dark, followed by the incubation with the addition of 100 mM DTT at RT. The urea concentration was reduced by diluting the mixture with MilliQ water, then the proteins digested with sequencing grade modified trypsin at 1:50 enzyme-to-substrate ratio. After 4 h of digestion at 37°C, samples were diluted 1:4 with 50 mM NH_4_HCO_3_, 1 mM CaCl_2_ and another aliquot of the same amount of trypsin was added to the samples and incubated at 37°C overnight. The digested samples were desalted on Sep-Pak C18 columns (Waters, Milford, MA, United States) and lyophilized for TMT labeling.

### Cell Lysis and Sample Preparation

Cells in pellet were washed with 1 mL of cold PBS to remove culture medium and any residual peptides. About 1 × 10^6^ cells were lysed with 300 μl of lysis buffer (8M urea, 0.8M NH_4_HCO_3_, pH 8.0) supplemented with 10 μl of protease inhibitor cocktail. The samples were then sonicated for 30 min on ice, and then centrifuged at 12,000 × *g* at 4°C for 15 min. The supernatants were collected, and protein concentration in the cell lysates was measured using the Pierce BCA protein assay on a NanoDrop 2000 (Thermo Fisher Scientific, Bremen, Germany). Then, 500 μg of protein was used for in-solution digestion as described above. Tryptic peptides were desalted on Sep-Pak C18 columns and lyophilized for TMT labeling.

### TMT-6plex Labeling for LC-MS/MS

TMT-labeling was carried out using the TMT 6plex labeling kit according to the manufacturer’s protocol. In brief, tryptic peptides resulting from 100 μg of protein per channel were used in the labeling. The TMT labeling reagents were dissolved in 41 μL acetonitrile per vial and added to the samples. For cell protein extracts, TMT-126, 127, 128, and 129 were used to label untreated, caerin 1.1, 1.9, and 1.1/1.9 treatments, respectively. For ESPs in cell environment, TMT-127, 128, 129, 130 were used to label untreated, caerin 1.1, 1.9, and 1.1/1.9 treatments, respectively. The reaction was incubated for 1 h at RT and quenched by 5% hydroxylamine. The labeled samples from the same biological replicate were mixed and desalted using Sep-Pak C18 columns and dried using a SpeedVac.

### uHPLC Tandem QTof MS/MS Analyses

TMT labeled peptides from cells or cell growth environment were resuspended in 25 μL of 0.1% formic acid in MilliQ water and analyzed by a QTOF X500R mass spectrometer (AB SCIEX, Concord, Canada) equipped with an electrospray ion source attached to an ExionLC liquid chromatography system (AB SCIEX, Concord, Canada). A 20 μL sample of each sample was injected into a 150 mm × 2.1 μm Agilent AdvanceBio Peptide Mapping column (Agilent Technologies, Mulgrave, VIC, Australia) equipped with a Fast Guard column for mass spectrometry analysis. Linear gradients of 5–60% solvent B over a 50 min period at a flow rate of 400 μL/min, followed by a gradient from 60 to 80% solvent B over 10 min and 80–95% solvent B in 5 min were used for peptide elution. Solvent B remained at 95% for a 1 min period for washing the column after which it was decreased to 5% for equilibration prior to the injection of the subsequent sample. Solvent A consisted of 0.1% formic acid in MilliQ water while solvent B contained 0.1% formic acid in 100% acetonitrile. The ion spray voltage was set to 5500 V, the declustering potential was set to 100 V, the curtain gas flow was set at 30, ion source gas 1 was set at 40, the ion source gas 2 was set at 50 and spray temperature was set at 450°C. The mass spectrometer acquired the mass spectral data in an Information Dependant Acquisition mode. Full scan TOFMS data was acquired over the mass range 350–1400 and for product ion ms/ms 50–1800. Ions observed in the TOF-MS scan exceeding a threshold of 100 cps and a charge state of +2 to +5 were set to trigger the acquisition of product ion. The data was acquired using SCIEX OS software (AB SCIEX, Concord, Canada).

### Protein Identification and Quantification

The LC-MS/MS data were converted using msconvert (Chambers et al., 2012) and imported to the PEAKS studio (Bioinformatics Solutions Inc., Waterloo, ON, Canada, version 7.0). The database of human proteome used was downloaded from Uniprot^1^ containing 70,613 entries. *De novo* sequencing of peptides, database search and characterizing specific PTMs were used to analyze the raw data; false discovery rate (FDR) was set to ≤1%, and [−10^∗^log(P)] was calculated accordingly where *P* is the probability that an observed match is a random event. The PEAKS used the following parameters: (i) precursor ion mass tolerance, 0.1 Da; (ii) fragment ion mass tolerance, 0.1 Da (the error tolerance); (iii) tryptic enzyme specificity with two missed cleavages allowed; (iv) monoisotopic precursor mass and fragment ion mass; (v) a fixed modification of cysteine carbamidomethylation; and (vi) variable modifications including TMT, lysine acetylation, deamidation on asparagine and glutamine, oxidation of methionine and conversion of glutamic acid and glutamine to pyroglutamate.

For the validation of quantification results, peptides with confidence ≥ 99% were used in PEAKS Q module. The mass error tolerance was set to 20 ppm, and the peptide score threshold (−10lgP) was 20. The results of differentially expressed proteins were validated sequentially by the following criteria, the proteins must contain at least one unique high confident peptide, the proteins have a *p-*values <0.05 and FDR ≤ 1%, and the fold change of proteins ≥ 1.5. A protein was included in the analysis when it was confidently identified in at least two biological replicates. The mass spectrometry proteomics data have been deposited to the ProteomeXchange Consortium via the PRIDE (Perez-Riverol et al., 2019) partner repository with the dataset identifier PXD015975.

### Western Blot

Caerin 1.1 and 1.9 mixture (molar ratio, 1:1) treated HeLa cells were diluted in SDS–PAGE sample buffer before mixed with loading buffer, electrophoresed through an 8% SDS–PAGE gel and then transferred to a PVDF membrane. The membrane was blocked with 5% skim milk in PBS and probed with different monoclonal antibodies (Abcam, ab202068 for caspase 9; Abcam, ab13847 for caspase 3; Abcam, #4249 for PI3K) at a dilution of 1:1000 respectively. Bound antibody was detected by incubation of the membrane with horseradish-peroxidase conjugated goat anti-rabbit/mouse antibody (PTG) at a dilution of 1:1000 and visualized using enhanced chemiluminescence (Fdbio science, Hangzhou, China) and visualized by an image scanner ProteinSimple (Santa Clara, CA, United States).

### Gene Ontology Analysis

The significantly up- or down- regulated proteins were subjected to gene ontology and pathway analysis using the online tool ToppFunc (Kaimal et al., 2010). ToppFunc adopts a hypergeometric model to evaluate the annotation frequency of an input gene list with respect to the one that randomly occurs. In the enrichment analyses of gene ontology, all the human genes in ToppFunc were used as background to calculate statistical significance. In addition, the Benjamini–Hochberg method implemented in ToppFunc was used to further exclude false negative results. A Bonferroni-corrected *P*-value < 0.05 was adopted as the cutoff for enriched biological processes.

### Protein–Protein Interaction (PPI) Analysis

Interactions among significantly regulated proteins were predicted using STRING (Szklarczyk et al., 2015). STRING provides a critical assessment and integration of protein-protein interactions from multiple resources, including direct (physical) as well as indirect (functional) associations. All resources were selected to generate the network and ‘confidence’ was used as the meaning of network edges and the required interaction score of 0.400 was selected for all PPI, except for the PPI of the treatment with caerin 1.1/1.9 (molar ratio 1:1), where a high confidence of 0.700 was used to highlight the most confident interactions. Neither the 1st nor 2nd shell of the PPI was included in this study. Protein without any interaction with other proteins was excluded from the network of this study.

### Pathway Analysis

The proteins determined to be differentially expressed with significance were analyzed by the online tool Innate DB^2^. The up-/down-regulation of key regulators identified, and their PPIs were used to predict the activation/inhibition of pathways. Significance of the activation or inhibition of pathways predicted by the analysis was tested by the Fisher Exact Test *P-*value, considering only the predictions with significant *P*-value of <0.05. Immune response and cancer signaling relevant pathways were focused in this study.

## Results

### Caerin 1.1 and 1.9 Inhibit Proliferation of HeLa Cells With Additive Effect

**FIGURE 1:**
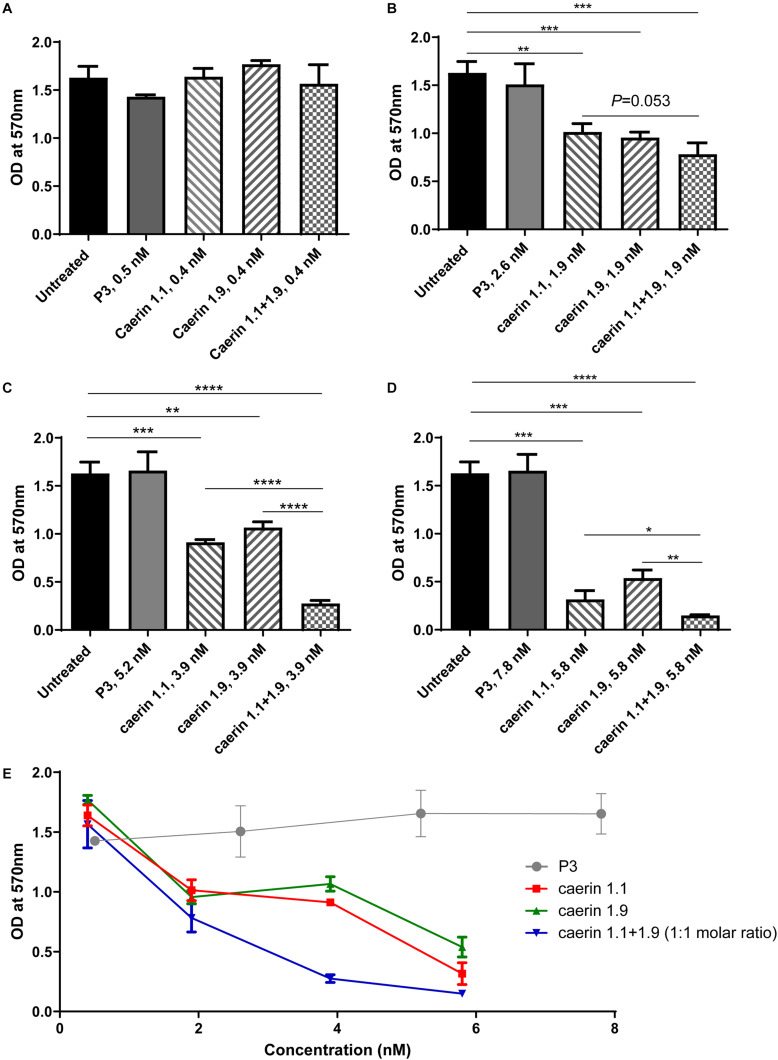
Caerin 1.1 and 1.9 inhibited the proliferation of HeLa cells by MTT assay. 1 × 10^4^ of HeLa cells were cultured in media only, or also with different concentrations of caerin 1.1, 1.9, P3 or the mixture of caerin 1.1/1.9 (molar ratio 1:1) for 24 h before MTT assay was performed. **(A)** 0.4 nM (0.5 nM for P3), **(B)** 1.9 nM (2.5 nM for P3), **(C)** 3.9 nM (5.2 nM for P3), **(D)** 5.8 nM (7.8 nM for P3), and **(E)** the dose dependency of activities. Each bar represents the statistical mean from three biological replicates (performed in triplicate) and the error bars represent the standard deviation. *P < 0.05, **P < 0.01, ***P < 0.001, ****P < 0.0001, two-tailed Student *T*-test.

For the MTT assays, cells without treatment and a peptide (P3) known to have no anti-proliferative effect (Ni et al., 2018) were used as negative controls (Figure 1). Triton X-100 (Anitha et al., 2012) and 31 nM caerin 1.1 were used as positive controls, both of which caused significant cell cytotoxicity (data not shown). None of the peptides showed any significant activity at 1 μg/ml (Figure 1A). With a concentration of 1.9 nM, both caerin 1.1 and 1.9 remarkably inhibited the proliferation of the cells. There appeared additive effect introduced by the treatment of caerin 1.1/1.9 (molar ratio 1:1) though it was insignificant (*P*-value = 0.0533) (Figure 1B). At the concentrations of 3.9 and 5.8 nM, not only did caerin 1.1 and 1.9 significantly inhibit the proliferation of cells, but also their mixture showed high additive effects compared to individual peptide (Figures 1C,D). Caerin 1.9 showed a higher activity at 3.9 and 5.8 nM with reference to caerin 1.1. Furthermore, their anti-proliferative activities were clearly dose dependent as shown in Figure 1E. The checkerboard assay found that most FICI values of the wells along the inhibition-no-inhibition boundary were less than 0.5, except one well with an FICI of 0.5900, and the average FICI was 0.4047 (one of triplicate assays showing consistent results was displayed in Supplementary Table 1). This suggested that caerin 1.1 and 1.9 have relatively high synergistic effect against HeLa cells. Our previous study indicated that neither caerin 1.1 nor 1.9, or their mixture inhibited the proliferation of non-cancer cells (incl. NP69 and HMC cells) at the similar concentrations that inhibited the growth of TC-1 cells (Ni et al., 2018).

### Caerin 1.1 and 1.9 Enter HeLa Cells With Different Magnitudes

**FIGURE 2:**
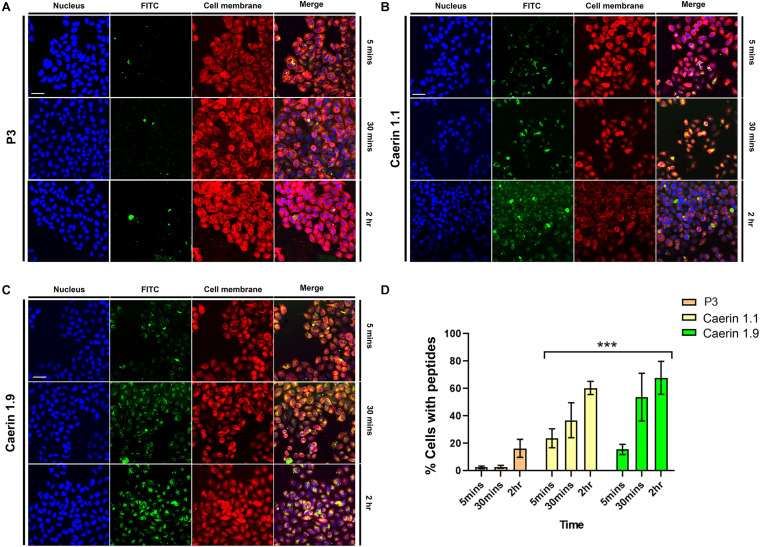
The co-localization of caerin 1.1, 1.9 and P3 in HeLa cells. HeLa cells were treated with FITC conjugated P3 (2.6 nM), caerin 1.1 and 1.9 (1.9 nM) for 5 min, 30 min, and 2 h. Then, cells were fixed by 4% paraformaldehyde and stained with Hoechst (Nucleus Marker, Blue) and CellMask (Cell membrane marker, Red). HeLa cells were treated with **(A)** P3, **(B)** caerin 1.1 and **(C)** caerin 1.9 for 5 min, 30 min, and 2 h. **(D)** The histogram shows the percentage of col-localization of P3, caerin 1.1, and 1.9 with HeLa cells. Data are shown as mean ± SEM. ***P < 0.001, two-way ANOVA, Tukey’s multiple comparisons test. Scale bar, 25 μm. The CellProfiler software was used to detect the co-localization of P3, caerin 1.1 and 1.9 with HeLa cells.

To reveal the process of caerin 1.1 and 1.9 interacting with cells, the cellular uptake of the peptides was assessed by confocal microscopy. Figure 2A shows weak fluorescence of P3 associated with cells at 5 and 30 min, implying a low level of uptake. Although relatively brighter green fluorescence of P3 can be observed at 2 h, it mainly distributed outside of the cells. There was bright green florescence present inside the cells and close to the nuclei (blue) treated by caerin 1.1 (Figure 2B) or 1.9 (Figure 2C) at 5 min, this was a sign of their internalization into the cells. This internalization increased with incubation time, as reflected by the increasing florescence intensity at 30 min and 2 h. Moreover, a clear overlap between the cell membrane and green florescence can be determined in the cells treated by caerin 1.1 for 2 h (Figure 2B), yet a similar consistency appeared at 30 min in the case of caerin 1.9 treatment (Figure 2C), which implies a stronger membrane/peptide interaction in the latter. At 2 h, there was a clear overlap between the distribution of caerin 1.9 and cell nuclei, compared to a lower extent in the treatment of caerin 1.1.

The quantitative analysis of cellular uptake based on the localization and intensity of fluorescence revealed that the locations of P3 on cell membrane were negligible at 5 and 30 min (Figure 2D) and cells with P3 increased to around 18% at 2 h. Approximately, 22 and 16% peptide-associated cells can be determined amongst the cells treated by caerin 1.1, or 1.9 respectively at 5 min, which largely increased to nearly 40 and 52% at 30 min. At 2 h post the treatment, there were about 60% of caerin 1.1 associated cells, compared to 70% in the case of caerin 1.9 treatment. The difference in the populations of peptide-associated cells with respect to caerin 1.1 and 1.9 were significant (*P*-value < 0.001) at each time point.

**FIGURE 3:**
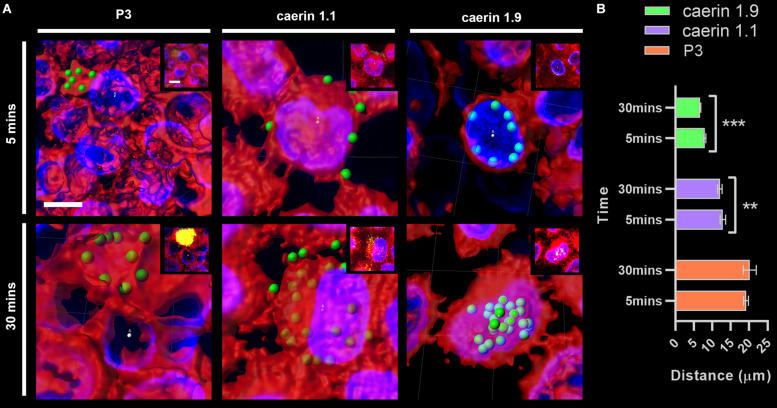
The distance between fluorescent signals and cell center points. The distance between P3, caerin 1.1 and 1.9 conjugated fluorescent signals with the cell center points was measured. **(A)** The distance between conjugated fluorescent signals with cell center points after treated by P3, caerin 1.1 and 1.9 for 5 min and 30 min. **(B)** The histogram shows the distance between the conjugated fluorescent signals with cell center points. Data are shown as mean ± SEM. ***P < 0.001, **P < 0.01, one-way ANOVA, Sidak’s multiple comparisons test. Scale bar, 10 μm. The images were captured by Confocal Laser Scanning Microscope (Olympus FLUOVIEW). The data three-dimensional reconstruction was generated by Imaris 7.

The distance between conjugated fluorescent signals of peptides and cell center points at 5 and 30 min were compared (Figure 3). P3 was basically distributed outside of cells (with an average distance of ∼20 μm) (Figure 3A) and the difference between two time points (i.e., 5 and 30 min) was insignificant (Figure 3B). Caerin 1.1 aggregated on the cell membrane at 5 min (distance, ∼14 μm), and moved closer (∼13 μm) to the center after 30 min (*P*-value < 0.01). The distances were much shorter in the case of caerin 1.9 that average signal already moved within 10 μm to the center at 5 min and decreased to 7.5 μm at 30 min (*P*-value < 0.001), indicating the potential distribution on the nucleus membrane. This also suggested that caerin 1.1 and 1.9 entered HeLa cells at different velocities.

We then explored the presence of unconjugated caerin 1.1 and 1.9 in cytoplasm of HeLa cells post the treatment, to further confirm the entry of caerin peptides into the cells and thus exclude the effect of FITC labeling toward the cellular uptake. The chromatograms of protein extract with a molecular weight below 10 kDa of caerin 1.1/1.9 treated HeLa cells at 1 and 2 h were compared to those of synthetic caerin peptides, where two chromatographic peaks emerging around the similar elution times of pure peptides were identified (Supplementary Figures 1A,D). Moreover, the MS/MS analysis of the 30s fraction including the peaks clearly identified the identities of caerin 1.1 and 1.9, respectively (Supplementary Figures 1E,F).

### Peptide Treatments Resulted Differential Protein Expressions in HeLa Cells and Cell Environment

**FIGURE 4:**
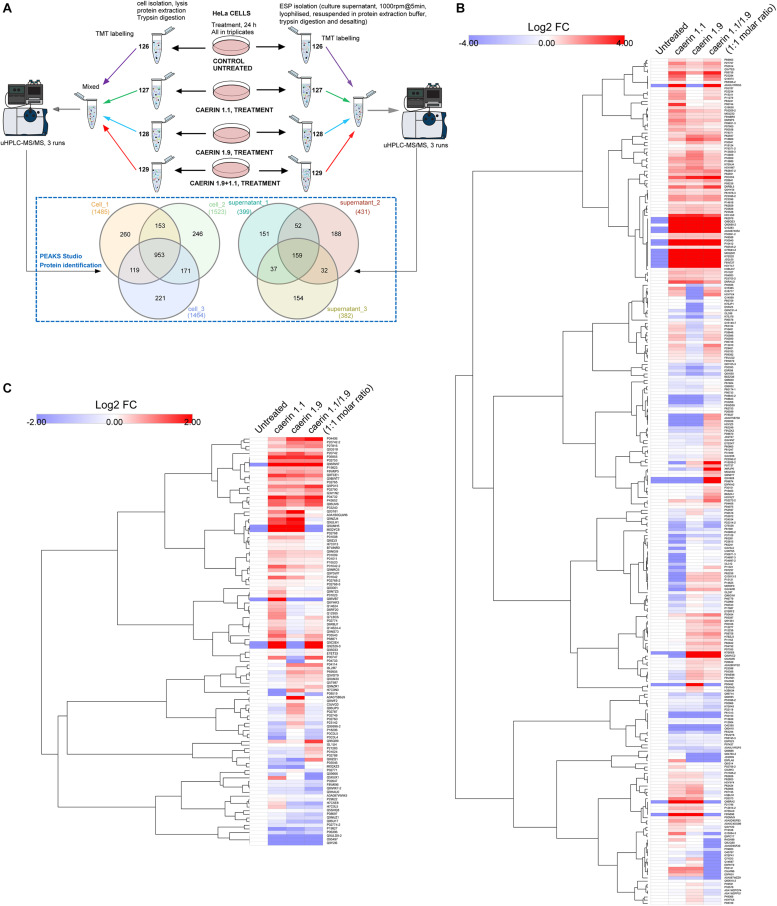
Quantitative proteomic analysis of the anti-proliferative activities caerin 1.1, 1.9 and their mixture (molar ratio 1:1) against breast cancer HeLa cells using quantitative proteomics. **(A)** Overall workflow for TMT labeling and uHPLC QTOF MS/MS analysis of cell and supernatant proteins/peptides extracted from untreated and peptide treated cells. The Venn diagram shows the comparison of proteins identified in biological triplicates of cell and cell supernatant samples (see Supplementary Tables 1, 2 for accessions of identified proteins, supporting peptides and protein annotations). **(B)** The hierarchy clusters of differentially expressed proteins (shown in Log2FC values) identified from cell samples (*n* = 3) post the peptide treatment (see Supplementary Figure 1–3 for proteins quantified in each replicate and Supplementary Table 3 for corresponding feature peptides). **(C)** The hierarchy clusters of differentially expressed proteins (shown in Log2FC values) identified from cell environment (supernatant) samples (*n* = 3) post the peptide treatment (see Supplementary Figures 4–6 for proteins quantified in each replicate and Supplementary Table 4 for corresponding feature peptides).

The workflow for TMT-labeling proteomic analysis is shown in Figure 4A, where cells were cultured with three treatments or untreated for 24 h in triplicates. The comparison of the proteins from the triplicates of the cells post the treatments resulted in a total of 953 proteins belonging to 234 protein groups were mutually identified with an FDR < 1% (Supplementary Table 2). There were 260, 246, and 221 proteins uniquely identified in each replicate. In addition, caerin 1.9 was present in all cell samples supported by high confidence MS/MS spectra of peptide segments, implying its potential association with cytoplasm or cell membrane (Supplementary Figures 2A–C); caerin 1.1 or P3 was not detected in these samples. In terms of proteins identified in cell environment, there were 159 proteins (50 protein groups) identified mutually from three replicates (Supplementary Table 3); only peptide segments of caerin 1.1 were detected (Supplementary Figures 2D–F).

The results of protein quantification and the feature peptides identified for each replicate of cell samples were recorded in Supplementary Table 4, with hierarchy clusters of differentially expressed proteins with significance were shown in Supplementary Figures 3–5. After the validation to include those showed consistent quantity variations in at least two replicates, a total of 267 protein groups were quantifiable out of proteins identified in cells (Figure 4A). In terms of the proteins identified in the cell environment, there were 112 protein groups taken into quantitation (Figure 4A and Supplementary Table 5). There were four protein groups mutually identified as quantifiable for cell and cell environment proteins, including glyceraldehyde-3-phosphate dehydrogenase, hemoglobin subunit alpha, neuroblast differentiation-associated protein and apolipoprotein A-I. The annotations of all quantifiable proteins were shown in Supplementary Table 6.

The quantitation results of cell proteins following treatments with caerin 1.1, 1.9 and 1.1/1.9 showed high level of similarity, yet differences can be observed (Figure 4B). With the treatment of caerin 1.1, the expressions of 100 protein groups showing significant upregulation (with fold change ≥ 1.5). These included nine proteins that were not identified in untreated cells, such as alpha-enolase, cyclin-dependent kinase inhibitor 2A, heterogeneous nuclear ribonucleoprotein L and calmodulin 3, which were also present with the treatments of caerin 1.9 and 1.1/1.9 except alpha-enolase (Supplementary Table 4). Several stress response related proteins were significantly upregulated, for instance, heat shock protein 90 (HSP90) beta family member 1, heat shock protein family B (small) member 1 and HSP60. Besides, a few enzymes, including enolase 1, prolyl 4-hydroxylase, cyclin dependent kinase 1, seryl-tRNA synthetase, glyoxalase I, aldolase, pyruvate kinase M1/2, creatine kinase, malate dehydrogenase 2, and so forth, showed remarkably higher expression levels (Supplementary Table 6). The treatments seemed to significantly reduce the abundance of structural proteins, such as actinin alpha 4, keratin 8, tubulin beta class I and actinin alpha 1.

With caerin 1.9 treatment, proteins such as poly(rC) binding protein 2, AHNAK nucleoprotein, peroxiredoxin 1, solute carrier family 25 member 5, peptidylprolyl isomerase A and calreticulin were significantly upregulated, while proteins differentially down-regulated included small nuclear ribonucleoprotein 200 kDa helicase, l-lactate dehydrogenase A chain, multifunctional protein ADE2, ribosomal protein L23 and so forth. In terms of caerin 1.1/1.9 treatment, upregulations of poly(rC) binding protein 3, ribosomal protein L19, serine/arginine rich splicing factor 2, protein disulfide isomerase family A member 3, solute carrier family 3 member 2 and CKB creatine kinase B showed upregulation (Supplementary Table 4).

In terms of proteins in cell growth environment shown in Figure 4C (the hierarchy clusters of differentially expressed proteins in each replicate were shown in Supplementary Figure 6–8), the NF-kappa-B signaling related protein, caspase recruitment domain family member 10 (CARD10) (Wang et al., 2001), was upregulated significantly post the caerin 1.1 treatment. Stress response related proteins showing elevated levels of expression included hemopexin, metallothioneins, tripartite motif containing 74 and sacsin. Three enzymes, plasmin, lysine demethylase and glyceraldehyde-3-phosphate dehydrogenase, were highly present. Complement C3, C4A and 4B were downregulated significantly by the treatment of caerin 1.1/1.9. The expressions of cytoskeletal proteins, including vinculin, fibulin 1, gelsolin and pleckstrin homology domain containing A5 were suppressed consistently by three treatments. A-kinase anchoring protein 9 and plasminogen were significantly elevated only by caerin 1.9.

### Enriched Biological Processes and Interactions of Significantly Upregulated Proteins

**FIGURE 5:**
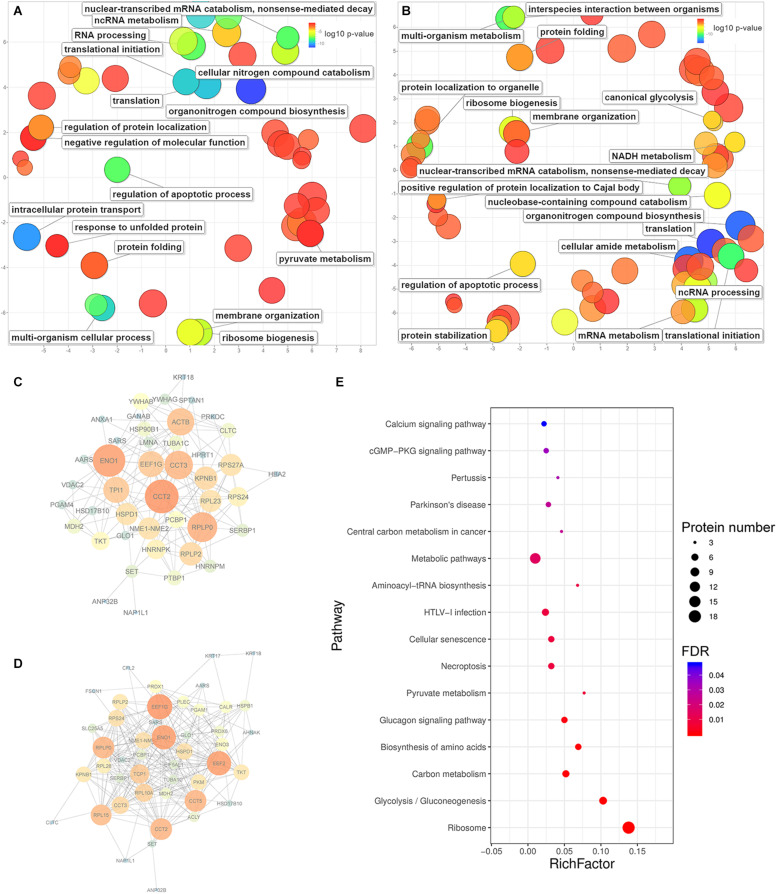
The enrichment of biological processes, protein-protein interactions and KEGG pathways predicted from significantly upregulated proteins identified in HeLa cells with the treatments of caerin 1.1 or 1.9. The enriched biological processes (Bonferroni-corrected *P*-values < 0.05) in HeLa cells with caerin 1.1 **(A)** and 1.9 **(B)** treatments; the PPI network predicted based on significantly regulated proteins induced by caerin 1.1 **(C)** and 1.9 **(D)** treatments; the KEGG pathways enriched by caerin 1.9 treatment **(E)** (see Supplementary Table 5 for all enriched processes, functions and cellular components).

The gene ontology enrichment of the significantly upregulated proteins was filtered by a Bonferroni-corrected *P-*value below 0.05. Most of the enriched biological process terms were similar with respect to caerin 1.1 or 1.9, such as organonitrogen compound biosynthesis, cellular protein localization, translation, protein transport, regulation of cell death and programmed cell death (Figures 5A,B). In cells treated by caerin 1.1, regulation of mRNA stability (corrected *P*-value = 8.25E-04), positive regulation of apoptotic process (3.34E-03), regulation of apoptotic signaling pathway (6.53E-03) and response to unfolded protein (4.03E-02) were enriched (Figure 5A). This contrasted caerin 1.9 treatment (Figure 5B), which shows enrichments in regulation of protein localization to Cajal body (9.50E-05), regulation of telomere maintenance via telomerase (2.04E-04), positive regulation of protein localization to nucleus (9.31E-04), positive regulation of cellular amide metabolic process (6.60E-03), and so forth (Supplementary Table 6). When treating the cells with caerin 1.1/1.9, the enrichments in the processes related to unfolded/incorrectly folded proteins were detected, such as response to topologically incorrect protein (3.50E-05), cellular protein-containing complex assembly (8.12E-04) and chaperone-mediated protein folding (4.26E-02) (Supplementary Figure 9). In addition, the cellular response to interleukin-4 became enriched (2.17E-03), so were responses to reactive oxygen species (7.40E-03) and oxidative stress (3.28E-02) (Supplementary Table 6).

The PPIs between significantly upregulated proteins due to the treatments of caerin 1.1 and 1.9 were shown in Figures 5C,D. Intensive interactions (enrichment *P*-value < 1.0E-16) involving 44 nodes with 173 edges were uncovered for the cell proteins post caerin 1.1 treatment. ENO1 and CCT2 showed the highest number of connections with other proteins, while RPLP0, EEF1G, ACTB, CCT3, KPNB1 and TPI1were also numerously connected (Figure 5C). The enrichment analysis of this PPI identified regulations of cell death and apoptotic processes as the two most enriched biological processes. In Figure 5D, there are 49 nodes connected by 263 edges, also presenting a highly enriched PPI (*P*-value < 1.0E-16). Similar nodes can be observed compared to those of caerin 1.1 treatment, such as ENO1, EEF1G, CCT2, RPLP0 and CCT5. Chaperone-mediated protein folding, translation and several biosynthetic and metabolic processes became highly enriched in Figure 5D. In terms of the PPIs of the dual treatment, there were additional highly connected protein families RPS and RPL (Supplementary Figure 9). Since more interactions were predicted among the proteins upregulated by caerin 1.9, the enrichment of KEGG pathways of these proteins were evaluated (Figure 5E). A few enriched pathways were disease-related, such as HTLV-I infection, central carbon metabolism in cancer, pertussis and Parkinson’s disease. The two most enriched pathways were calcium and cGMP-PKG signaling pathways.

For the proteins identified in the cell growth environment post the treatment of caerin 1.1, a few enzyme-related biological processes were significantly enriched, including negative regulations of endopeptidase activity (corrected *P*-value = 1.93E-04), peptidase activity (2.67E-04), proteolysis (2.86E-03) and response to wounding (1.80E-02) (Supplementary Table 7). In terms of caerin 1.9 treatment, the enrichment analysis found blood coagulation and fibrin clot formation as the most significant term (4.06E-03) for upregulated proteins. Significantly less complex PPIs were identified compared to those of cells (Supplementary Figure 10). HPX and PLG were the nodes highly interacting with other proteins due to the caerin 1.1 treatment, while KNG1, ITIH2 and SERPINC1 were exceedingly connected in the treatment of caerin 1.9, indicating APOB could be its potential target (Supplementary Figures 10B,C).

### Caerin 1.1 and 1.9 Treatment Stimulated TNF-α Dependent Apoptotic Signaling in HeLa Cells

**FIGURE 6:**
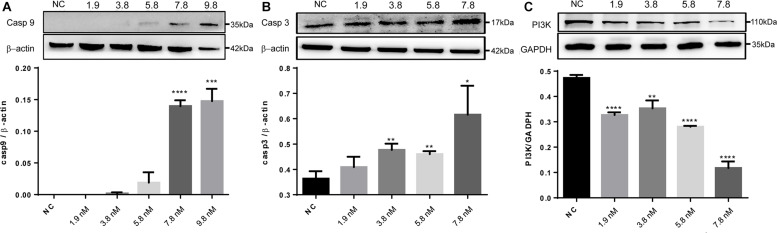
Western blot analysis of CASP9, CASP3 and PI3K of HeLa cells after treated by caerin 1.1/1.9. 5 × 10^5^ cells were either untreated or treated with different concentrations (5, 10, 15, 20, and 25 μg/ml) of caerin 1.1/1.9 mixture (mass ratio 50:50) for 24 h. The gel lane areas corresponding to the molecular weights of target proteins and β-actin/GAPDH were cropped and subjected to the first antibody treatment, respectively. Bound antibody was detected by incubation of the membrane with horseradish-peroxidase conjugated goat anti-rabbit/mouse antibody (PTG) at a dilution of 1:1000 and visualized using enhanced chemiluminescence and visualized by an image scanner ProteinSimple. The experiment was performed in triplicate and one representative blot was quantified, **(A)** CASP9, **(B)** CASP3, and **(C)** PI3K, with the original gel images shown in Supplementary Figure 13. A two-tailed Student *t*-test was used to calculate *P*-values: *P < 0.05, **P < 0.01, ***P < 0.001, ****P < 0.0001.

The significantly regulated proteins were further assessed to characterize potential signaling pathways that were enhanced or suppressed in response to the treatments. With the cell proteins identified following caerin 1.1 treatment, there were four pathways showing significance (with a corrected *P*-value < 0.05), including EGFR1 (corrected *P*-value < 1E-5), androgen receptor (0.00002), TNF-α (0.00015) and TCR (0.00038) pathways, among which EGFR1 pathway was also identified as significantly modulated in cell growth environment. Based on the quantitation of cell proteins (Supplementary Table 4) and PPIs, it was indicative that apoptosis section of TNF-α pathway was activated due to the treatments of caerin 1.1 and 1.9 (Supplementary Figure 11), which was confirmed by the dose-dependent elevated levels of CASP9 and CASP3 (Figures 6A,B). The inactivation of EGFR was displayed in Supplementary Figure 12, which suggested the downregulation of AKT1, a sign that PI3K/AKT signaling could be potentially inhibited, confirmed by decreasing expression level of PI3K as shown in Figure 6C (the original membrane images of western blot results were displayed in Supplementary Figure 13). In addition to the pathways modulated by caerin 1.1, TSLP and α_6_β_4_ signaling pathways were also detected with significance in the treatments of caerin 1.9 and 1.1/1.9.

## Discussion

Frog skin peptides as promising agents to treat infections produced by drug-resistant microorganisms have been suggested by many reports, and the mechanism appeared relevant to the modulation of inflammatory signaling pathways (Conlon et al., 2019). Our previous study has shown that caerin 1.1 and 1.9 significantly inhibited the proliferation of TC-1 cells with dose-dependent additive effect *in vitro* but had no observable effects on a few normal human cell lines (8). They seemed to cause the release of acute inflammatory response cytokines and chemokines, suggesting the potential to improve the immune suppressive environment (8). In this study, the downregulation of structural/cytoskeletal proteins was observed, implying the integrity of the cells was impacted by the peptide treatments, which could limit the migration and metastasis tumor cells (Fife et al., 2014). A previous study showed that caerin peptides were able to access HIV captured by dendritic cells either on the cell surface or intracellularly, thus effectively in inhibiting HIV infection by disrupting the viral membrane and preventing the entry the transfer of HIV to T cells (VanCompernolle et al., 2005). This was indicative by the co-localization between caerin 1.1 or 1.9 and HeLa cell membrane and the consequent internalization of these peptides within about 5 min into the treatment (Figure 2), which was confirmed by the identification of these peptides in cytoplasm of treated HeLa cells (Supplementary Figure 1). In contrast to the presence of caerin 1.1 in the cell growth environment, the absence of caerin 1.9 might be related to its higher magnitude of cellular uptake; this agreed with the observation that conjugated fluorescence signal of caerin 1.9 increased within cells more rapidly compared to caerin 1.1 and P3.

It was found that keratin 8 depleted cells exhibited high level of apoptosis (Fortier et al., 2013); the suppression of karatin 8 by caerin 1.1 and 1.9 could contribute to the apoptosis of HeLa cells (Supplementary Table 5), as reflected by the enrichments of positive regulation of apoptotic process (corrected *P*-value = 3.34E-03) and regulation of apoptotic signaling pathway (corrected *P*-value = 6.53E-03). This was verified by the western blot, where both CASP3 and 9 showed a higher expression levels with the presence of the caerin 1.1/1.9 mixture (Figure 6). Moreover, a few serpin subfamilies showed significant upregulations post the peptide treatments, which potentially activate hormone transport (SERPINA7), coagulation and angiogenesis (SERPINC1 and F1) (Heit et al., 2013). Chaperonin containing TCP1 (CCT) was previously identified as the target of a cancer-specific cytotoxic peptide, and increased level of CCT was observed in cancer cells susceptible to the peptide (Bassiouni et al., 2016). In our study, CCT was highly present in caerin 1.1 and caerin 1.1/1.9 treatments, suggesting its potential relevance with caerin 1.1.

The peptide treatments remarkably suppressed the expressions of a few enzymes appeared to be more active in cancers, for example, triosephosphate isomerase (TPI), argininosuccinate synthase 1 (ASS1), carbamoyl-phosphate synthase 1 (CPS1), aldo-keto reductase family 1 member B10 (AKR1B10), lactate dehydrogenase B (LDHB) and phosphoglycerate kinase 1 (PGK1). TPI plays roles in migration and invasion of cancer cells (Altenberg and Greulich, 2004) and is correlated with the anti-drug resistance in human gastric cancer cells (Wang et al., 2008). ASS1 has been suggested as an upregulated target for primary human colorectal tumors, whose inhibition or genetic ablation potentially allows colorectal cancer pathogenicity (Bateman et al., 2017). CPS1, regulated by MAP kinase (Graves et al., 2000), exhibits increased activities in liver cancer (Aoki and Weber, 1981). Significant correlation has been identified between overexpression of AKR1B10 and the proliferation of different types of cancer cells (Fukumoto et al., 2005; Jin et al., 2006, 2016; Wang et al., 2009). LDHB is largely used by cancer cells to bypass oxidative phosphorylation process to produce lactate directly from pyruvate to promote the cell proliferation (Vander Heiden et al., 2009; Dennison et al., 2013), while transactivated by the key tumorigenic driver, signal transducer and activator of transcription 3 (STAT3) (Zha et al., 2011). The overexpression of PGK1 has been found to not only promote the proliferation of cancer cells (Wang et al., 2010; Zieker et al., 2010; Ahmad et al., 2013), but also relate to multidrug resistance phenotype (Duan et al., 2002; Sun et al., 2015). Meanwhile, these enzymes have key functions in glycolytic metabolism or urea cycle, the significant suppression of them, due to caerin 1.1/1.9 treatment, strongly suggest negative impacts on the proliferation of HeLa cells. The elevation of creatine kinase (Yan, 2016) and a few heat shock protein subfamilies (Wettstein et al., 2012) also indicated the cells retained a stressed state post the treatment. In addition, the treatments also consistently increased the levels of a few ribosomal proteins, which suggested impacts on biological processes of translation, RNA processing, protein localization and ribosome biogenesis, as reflected in Figure 5.

Accelerated receptor recycling and increased excretion/secretion of matrix components, adhesion molecules and growth factors usually accompany the rapid proliferation of cancer cells (Hu and Polyak, 2008), which was reflected in our observation that intracellular and membrane-associated proteins were identified with high abundance in cell growth environment, such as MRPL15, PPARD, MAP4 and OR5A2. The upregulation of MAP3K12 binding inhibitory protein 1 (MBIP) induced by caerin 1.1 and 1.1/1.9 treatments could consequently activate the SAPK/JNK signaling pathway to consequently trig apoptosis (Nishina et al., 2004). The nucleation-promoting protein gelsolin was suppressed in all treatments, indicating the assembly filaments were deactivated (Sun et al., 1999) and an potential enhancement of apoptosis since cells express an increased level of gelsolin to counteract apoptosis (Koya et al., 2000).

Many studies have reported that TNF-α pathway plays important roles in regulating tumor proliferation, migration, invasion and angiogenesis (Balkwill, 2009), its aberrantly expression poses consequential impact on the TME (Landskron et al., 2014). Thus, the possible activation of apoptosis in TNF-α signaling might further alter the TME (Supplementary Figure 11). It has been found that EGFR pathway, working collaboratively with the PI3K/PTEN/AKT/mTORC1 pathway, play prominent roles in the development of breast cancer (Davis et al., 2014), prognosis and survival of breast cancer patients (Witton et al., 2003), which means the inhibition of EGFR signaling by the peptides could suppress malignant transformation and build up apoptosis. The upregulation of ANXA1 detected due to the peptide treatments could potentially cause the inhibition (Supplementary Figure 12), since ANXA1 negatively correlates with the activation of EGFR signaling via the EGF stimulated EGFR tyrosin 1068 phosphorylation and its downstream AKT activity (Raulf et al., 2018).

The signaling of androgen receptor is related to the progression of prostate cancer (Attard et al., 2011) and considered as a therapeutical target for estrogen receptor-negative breast cancer (Ni et al., 2011). It was inhibited by the treatment of caerin 1.1 or 1.9 via the regulators on the pathway, such as CALR, CDK1, FLNA, GAPDH, GNB2L1, HSP90AA1, HSP90B1, and HSPA5. Signal transduction through the TCR pathway is essentially required for T cells to enter the cell cycle and proliferate (Foell et al., 2007), possibly via costimulation of TCR and CD28 pathways (Hanley et al., 2008). This means that the activation of TCR pathway induced by caerin 1.1 or 1.9 might result in T cells stimulation, which well accords with our recent investigation that caerin peptides can recruit significant more T cells to the tumor site using a mouse model (Pan et al., 2019). TSLP pathway has been found to relate to the progression of several human cancers, including breast, pancreatic, gastric and cervical cancer, as well as B cell lymphoma and myeloma (De Monte et al., 2011; Xie et al., 2013; Nakajima et al., 2014; Barooei et al., 2015), via generating type 2–biased inflammation in the tumor microenvironment (Pedroza-Gonzalez et al., 2011). However, one investigation claimed that no meaningful correlation has been identified between TSLP pathway activity and breast cancer (Ghirelli et al., 2016). The signal transduction of α6β4 pathway works together with EGFR clustering to promote tumor cell motility and invasion of breast cancer (Gilcrease et al., 2009). The activation α6β4 pathway might be the response of cells tackling the apoptotic stress induced by the peptides. Our study suggested here that caerin 1.1 and 1.9 were able to stimulate TNFα-dependent apoptosis in HeLa cells, as well as module several important innate immune signaling via altering the expressions of protein regulators on the pathways. Thus, the applications of these peptides together with therapeutic vaccine could potentially improve the TME to provide a higher anti-tumor efficacy.

## Conclusion

The host-defense natural peptides caerin 1.1, 1.9 and their mixture (molar ratio 1:1) inhibited the proliferation of HeLa cells *in vitro* with dose-dependent activity. The interaction between the peptides and nucleus membrane was suggested. The TMT-labeling proteomics revealed significantly different protein profiles of cells and cell growth environment 24 h post the treatment. Biological processes including translation, apoptosis, glycolytic metabolism, protein folding, and localization were enriched in cells, while downregulations of endopeptidase and peptidase activities were highly representative in cell growth environment. The activation of apoptotic signaling was supported by differentially expressed proteins of both cells and the environment, which might be executed via TNF-α signaling pathway as confirmed by western blot. Meanwhile, the recruitment of T cells to the cell growth environment was indicative since the TCR pathway appeared significantly more activated. Thus, the applications of these peptides together with therapeutic vaccine might potentially improve the TME to provide a higher anti-tumor efficacy.

## Data Availability Statement

The datasets presented in this study can be found in online repositories. The names of the repository/repositories and accession number(s) can be found below: https://www.ebi.ac.uk/pride/archive/, PXD015975.

## Author Contributions

TW and XL: conceptualization, methodology, writing—original draft preparation, funding acquisition, and supervision. TW and MC: software. BY and GN: validation. GN, SC, MC, and JW: formal analysis and data curation. JY, SW, HL, and YW: investigation. MW and GC: resources. TW, XL, and SW: writing—review and editing. MC, TW, and XL: visualization. SC and YW: project administration. All authors read and agreed to the published version of the manuscript.

## Conflict of Interest

The authors declare that the research was conducted in the absence of any commercial or financial relationships that could be construed as a potential conflict of interest.

## Footnotes

1 http://www.uniprot.org/proteomes/UP000005640

2 https://www.innatedb.ca/index.jsp

## Supplementary Material

FIGURE S1 Identification of caerin 1.1 and 1.9 in the cytoplasm of HeLa cells. The chromatograms of synthetic caerin 1.1 **(A)**, caerin 1.9 **(B)**, cytoplasmic protein extracts (MW <10kDa) of HeLa cells treated by 3.9 nM caerin 1.1/1.9 (1:1 molar ration) at 1 hr **(C)** and 2 hr **(D)**. Arrows indicate the peaks potentially corresponding to caerin 1.1 or 1.9, respectively. The selected MS/MS spectra of caerin 1.1 **(E)** and 1.9 **(F)** identified in the 30s fraction collected around the potential peaks in cytoplasm of HeLa cells.

FIGURE S2 Identification of caerin 1.9 in HeLa cells and caerin 1.1 in cell environment post TMT labelling. The coverage of caerin 1.9 sequence and MS/MS spectra of supporting peptides in cell samples, **(A)** replicate 1, **(B)** replicate 2 and **(C)** replicate 3. The coverage of caerin 1.1 sequence and MS/MS spectra of supporting peptides in cell growth environment, **(D)** replicate 1, **(E)** replicate 2 and **(F)** replicate 3.

FIGURE S3 Hierarchy cluster of differentially expressed proteins with significance (fold change = 1.5) in HeLa cells, identified from TMT labelling analysis of biological replicate 1 treated with caerin 1.1, 1.9 and 1.1/1.9 with reference to untreated cells at 24 h. The magnitudes of up-/down- regulation (Log2FC values) are indicated by color change. The fold change data was listed in Supplementary Table 4.

FIGURE S4 Hierarchy cluster of differentially expressed proteins with significance (fold change = 1.5) in HeLa cells, identified from TMT labelling analysis of biological replicate 2 treated with caerin 1.1, 1.9 and 1.1/1.9 with reference to untreated cells at 24 h. The magnitudes of up-/down- regulation (Log2FC values) are indicated by color change. The fold change data was listed in Supplementary Table 4.

FIGURE S5 Hierarchy cluster of differentially expressed proteins with significance (fold change = 1.5) in HeLa cells, identified from TMT labelling analysis of biological replicate 3 treated with caerin 1.1, 1.9 and 1.1/1.9 with reference to untreated cells at 24 h. The magnitudes of up-/down- regulation (Log2FC values) are indicated by color change. The fold change data was listed in Supplementary Table 4.

FIGURE S6 Hierarchy cluster of differentially expressed proteins with significance (fold change = 1.5) in the growth environment of HeLa cells, identified from TMT labelling analysis of biological replicate 1 treated with caerin 1.1, 1.9 and 1.1/1.9 with reference to those of untreated cells at 24 h. The magnitudes of up-/down- regulation (Log2FC values) are indicated by color change. The fold change data was listed in Supplementary Table 5.

FIGURE S7 Hierarchy cluster of differentially expressed proteins with significance (fold change = 1.5) in the growth environment of HeLa cells, identified from TMT labelling analysis of biological replicate 2 treated with caerin 1.1, 1.9 and 1.1/1.9 with reference to those of untreated cells at 24 h. The magnitudes of up-/down- regulation (Log2FC values) are indicated by color change. The fold change data was listed in Supplementary Table 5.

FIGURE S8 Hierarchy cluster of differentially expressed proteins with significance (fold change = 1.5) in the growth environment of HeLa cells, identified from TMT labelling analysis of biological replicate 3 treated with caerin 1.1, 1.9 and 1.1/1.9 with reference to those of untreated cells at 24 h. The magnitudes of up-/down- regulation (Log2FC values) are indicated by color change. The fold change data was listed in Supplementary Table 5.

FIGURE S9 Enriched biological processes and PPIs of significantly upregulated proteins in cells treated with the mixture of caerin 1.1 and 1.9 (mass ratio 50:50).

FIGURE S10 The enriched PPIs of proteins identified in the growth environment of HeLa cells with the peptide treatments.

FIGURE S11 Caerin 1.1/1.9 treatment modulated TNFα signalling pathway. TNFα signalling pathway was identified as the canonical pathway in Hela cells significantly affected by the treatment of caerin 1.1/1.9, based on TMT-labelling data. Lines connecting the proteins represent known interactions, and arrows indicate the directions of up-/down-stream regulations. The pathway was downloaded and modified from WikiPathways (https://www.wikipathways.org/index.php/Pathway:WP231).

FIGURE S12 Caerin 1.1/1.9 treatment modulated EGFR signalling pathway. EGFR signalling pathway was identified as the canonical pathway in Hela cells significantly affected by the treatment of caerin 1.1/1.9, based on TMT-labelling data. Lines connecting the proteins represent known interactions, and arrows indicate the directions of up-/down-stream regulations. The pathway was downloaded and modified from WikiPathways (https://www.wikipathways.org/index.php/Pathway:WP437).

FIGURE S13 The original membrane images of western blot results shown in Figure 6, visualised by the image scanner ProteinSimple. From left to right, the blots corresponded to NC, 5, 10, 15, 20, and 25 μg/ml of peptide treatments, respectively.

TABLE S1 The calculation of IC50 values of caerin 1.1, 1.9 and 1.1/1.9 (1:1 molar ratio) and their synergistic effect evaluated by the checkerboard assay.

TABLE S2 Protein identification results of three biological replicates of HeLa cells treated by caerin 1.1., 1.9 and 1.1/1.9. For each replicate, there are protein identified and supporting peptides.

TABLE S3 Protein identification results of three biological replicates of HeLa cell growth environment treated by caerin 1.1., 1.9 and 1.1/1.9. For each replicate, there are protein identified and supporting peptides.

TABLE S4 Protein quantitation results of three biological replicates of HeLa cells treated by caerin 1.1., 1.9 and 1.1/1.9. For each replicate, there are protein identified and supporting peptides. For each replicate, there are all TMT labelled proteins and significantly regulated proteins.

TABLE S5 Protein quantitation results of three biological replicates of HeLa cell growth envrionment treated by caerin 1.1., 1.9 and 1.1/1.9. For each replicate, there are protein identified and supporting peptides. For each replicate, there are all TMT labelled proteins and significantly regulated proteins.

TABLE S6 The enrichments of biological processes and pathways of significantly regulated proteins identified in HeLa cells post the treatments.

TABLE S7 The enrichments of biological processes and pathways of significantly regulated proteins identified in HeLa cell environment post the treatments.

## References

[B2] Ahmad S. S. Glatzle J. Bajaeifer K. Buhler S. Lehmann T. Konigsrainer I. (2013). Phosphoglycerate kinase 1 as a promoter of metastasis in colon cancer. *Int. J. Oncol.* 43 586–590. 10.3892/ijo.2013.1971 23727790

[B3] Altenberg B. Greulich K. O. (2004). Genes of glycolysis are ubiquitously overexpressed in 24 cancer classes. *Genomics* 84 1014–1020. 10.1016/j.ygeno.2004.08.010 15533718

[B4] Anitha A. Maya S. Deepa N. Chennazhi K. P. Nair S. V. Jayakumar R. (2012). Curcumin-loaded N,O-carboxymethyl chitosan nanoparticles for cancer drug delivery. *J. Biomater. Sci. Polym. Edn.* 23 1381–1400. 10.1163/092050611x581534 21722423

[B5] Aoki T. Weber G. (1981). Carbamoyl phosphate synthetase (glutamine-hydrolyzing): increased activity in cancer cells. *Science* 212 463–465. 10.1126/science.7209543 7209543

[B6] Attard G. Richards J. De Bono J. S. (2011). New strategies in metastatic prostate cancer: targeting the androgen receptor signaling pathway. *Clin. Cancer Res.* 17 1649–1657. 10.1158/1078-0432.ccr-10-0567 21372223 PMC3513706

[B7] Balkwill F. (2009). Tumour necrosis factor and cancer. *Nat. Rev. Cancer* 9 361–371. 10.1038/nrc2628 19343034

[B8] Barooei R. Mahmoudian R. A. Abbaszadegan M. R. Mansouri A. Gholamin M. (2015). Evaluation of thymic stromal lymphopoietin (TSLP) and its correlation with lymphatic metastasis in human gastric cancer. *Med. Oncol.* 32:217. 10.1007/s12032-015-0653-4 26175262

[B9] Bassiouni R. Nemec K. N. Iketani A. Flores O. Showalter A. Khaled A. S. (2016). Chaperonin containing TCP-1 protein level in breast cancer cells predicts therapeutic application of a cytotoxic peptide. *Clin. Cancer Res.* 22 4366–4379. 10.1158/1078-0432.ccr-15-2502 27012814 PMC5010522

[B10] Bateman L. A. Ku W. M. Heslin M. J. Contreras C. M. Skibola C. F. Nomura D. K. (2017). Argininosuccinate synthase 1 is a metabolic regulator of colorectal cancer pathogenicity. *ACS Chem. Biol.* 12 905–911. 10.1021/acschembio.6b01158 28229591

[B11] Chambers M. C. Maclean B. Burke R. Amodei D. Ruderman D. L. Neumann S. (2012). A cross-platform toolkit for mass spectrometry and proteomics. *Nat. Biotechnol.* 30 918–920. 23051804 10.1038/nbt.2377 PMC3471674

[B12] Chau B. N. Chen T. T. Wan Y. Y. Degregori J. Wang J. Y. (2004). Tumor necrosis factor alpha-induced apoptosis requires p73 and c-ABL activation downstream of RB degradation. *Mol. Cell Biol.* 24 4438–4447. 10.1128/mcb.24.10.4438-4447.2004 15121862 PMC400462

[B13] Conlon J. M. Mechkarska M. Leprince J. (2019). Peptidomic analysis in the discovery of therapeutically valuable peptides in amphibian skin secretions. *Expert. Rev. Proteom.* 16 897–908. 10.1080/14789450.2019.1693894 31729236

[B14] Davis N. M. Sokolosky M. Stadelman K. Abrams S. L. Libra M. Candido S. (2014). Deregulation of the EGFR/PI3K/PTEN/Akt/mTORC1 pathway in breast cancer: possibilities for therapeutic intervention. *Oncotarget* 5 4603–4650. 10.18632/oncotarget.2209 25051360 PMC4148087

[B15] De Monte L. Reni M. Tassi E. Clavenna D. Papa I. Recalde H. (2011). Intratumor T helper type 2 cell infiltrate correlates with cancer-associated fibroblast thymic stromal lymphopoietin production and reduced survival in pancreatic cancer. *J. Exp. Med.* 208 469–478. 10.1084/jem.20101876 21339327 PMC3058573

[B16] Deng Y. Ren X. Yang L. Lin Y. Wu X. (2003). A JNK-dependent pathway is required for TNFalpha-induced apoptosis. *Cell* 115 61–70. 10.1016/s0092-8674(03)00757-8 14532003

[B17] Dennison J. B. Molina J. R. Mitra S. Gonzalez-Angulo A. M. Balko J. M. Kuba M. G. (2013). Lactate dehydrogenase B: a metabolic marker of response to neoadjuvant chemotherapy in breast cancer. *Clin. Cancer Res.* 19 3703–3713. 10.1158/1078-0432.ccr-13-0623 23697991 PMC3727144

[B18] Diesendruck Y. Benhar I. (2017). Novel immune check point inhibiting antibodies in cancer therapy-opportunities and challenges. *Drug Resist. Updat.* 30 39–47. 10.1016/j.drup.2017.02.001 28363334

[B19] Duan Z. Lamendola D. E. Yusuf R. Z. Penson R. T. Preffer F. I. Seiden M. V. (2002). Overexpression of human phosphoglycerate kinase 1 (PGK1) induces a multidrug resistance phenotype. *Anticancer Res.* 22 1933–1941. 12174867

[B20] Fife C. M. Mccarroll J. A. Kavallaris M. (2014). Movers and shakers: cell cytoskeleton in cancer metastasis. *Br. J. Pharmacol.* 171 5507–5523. 10.1111/bph.12704 24665826 PMC4290699

[B21] Foell J. Hewes B. Mittler R. S. (2007). T cell costimulatory and inhibitory receptors as therapeutic targets for inducing anti-tumor immunity. *Curr. Cancer Drug Targets* 7 55–70. 10.2174/156800907780006841 17305478

[B22] Fortier A. M. Asselin E. Cadrin M. (2013). Keratin 8 and 18 loss in epithelial cancer cells increases collective cell migration and cisplatin sensitivity through claudin1 up-regulation. *J. Biol. Chem.* 288 11555–11571. 10.1074/jbc.m112.428920 23449973 PMC3630871

[B23] Fukumoto S. Yamauchi N. Moriguchi H. Hippo Y. Watanabe A. Shibahara J. (2005). Overexpression of the aldo-keto reductase family protein AKR1B10 is highly correlated with smokers’ non-small cell lung carcinomas. *Clin. Cancer Res.* 11 1776–1785. 10.1158/1078-0432.ccr-04-1238 15755999

[B24] Galliverti G. Tichet M. Domingos-Pereira S. Hauert S. Nardelli-Haefliger D. Swartz M. A. (2018). Nanoparticle conjugation of human papillomavirus 16 E7-long peptides enhances therapeutic vaccine efficacy against solid tumors in mice. *Cancer Immunol. Res.* 6:166. 10.1158/2326-6066.CIR-18-0166 30131378

[B25] Ghirelli C. Sadacca B. Reyal F. Zollinger R. Michea P. Sirven P. (2016). No evidence for TSLP pathway activity in human breast cancer. *Oncoimmunology* 5:e1178438. 10.1080/2162402x.2016.1178438 27622057 PMC5007973

[B26] Gilcrease M. Z. Zhou X. Lu X. Woodward W. A. Hall B. E. Morrissey P. J. (2009). Alpha6beta4 integrin crosslinking induces EGFR clustering and promotes EGF-mediated Rho activation in breast cancer. *J. Exp. Clin. Cancer Res.* 28:67. 10.1186/1756-9966-28-67 PMC2694164 19470173

[B27] Graves L. M. Guy H. I. Kozlowski P. Huang M. Lazarowski E. Pope R. M. (2000). Regulation of carbamoyl phosphate synthetase by MAP kinase. *Nature* 403 328–332. 10.1038/35002111 10659854

[B28] Hanley C. Layne J. Punnoose A. Reddy K. M. Coombs I. Coombs A. (2008). Preferential killing of cancer cells and activated human T cells using ZnO nanoparticles. *Nanotechnology* 19:295103 10.1088/0957-4484/19/29/295103 PMC2558672 18836572

[B29] Heit C. Jackson B. C. Mcandrews M. Wright M. W. Thompson D. C. Silverman G. A. (2013). Update of the human and mouse SERPIN gene superfamily. *Hum. Genom.* 7:22. 10.1186/1479-7364-7-22 24172014 PMC3880077

[B30] Hsieh M. H. Yu C. M. Yu V. L. Chow J. W. (1993). Synergy assessed by checkerboard. A critical analysis. *Diagn. Microbiol. Infect. Dis.* 16 343–349. 10.1016/0732-8893(93)90087-n 8495592

[B31] Hu M. Polyak K. (2008). Microenvironmental regulation of cancer development. *Curr. Opin. Genet. Dev.* 18 27–34. 10.1016/j.gde.2007.12.006 18282701 PMC2467152

[B32] Jin J. Krishack P. A. Cao D. (2006). Role of aldo-keto reductases in development of prostate and breast cancer. *Front. Biosci.* 11 2767–2773. 10.2741/2006 16720349

[B33] Jin J. Liao W. Yao W. Zhu R. Li Y. He S. (2016). Aldo-keto reductase family 1 member B 10 mediates liver cancer cell proliferation through sphingosine-1-phosphate. *Sci. Rep.* 6:22746. 10.1038/srep22746 PMC4780005 26948042

[B34] Kaimal V. Bardes E. E. Tabar S. C. Jegga A. G. Aronow B. J. (2010). ToppCluster: a multiple gene list feature analyzer for comparative enrichment clustering and network-based dissection of biological systems. *Nucleic Acids Res.* 38 W96–W102. 20484371 10.1093/nar/gkq418 PMC2896202

[B35] Koya R. C. Fujita H. Shimizu S. Ohtsu M. Takimoto M. Tsujimoto Y. (2000). Gelsolin inhibits apoptosis by blocking mitochondrial membrane potential loss and cytochrome c release. *J. Biol. Chem.* 275 15343–15349. 10.1074/jbc.275.20.15343 10809769

[B36] Landskron G. De La Fuente M. Thuwajit P. Thuwajit C. Hermoso M. A. (2014). Chronic inflammation and cytokines in the tumor microenvironment. *J. Immunol. Res.* 2014:149185. 10.1155/2014/149185 PMC4036716 24901008

[B37] Leo P. J. Madeleine M. M. Wang S. Schwartz S. M. Newell F. Pettersson-Kymmer U. (2017). Defining the genetic susceptibility to cervical neoplasia-A genome-wide association study. *PLoS Genet.* 13:e1006866. 10.1371/journal.pgen.1006866 28806749 PMC5570502

[B38] Ma B. Yuan J. Chen S. Huang K. Wang Q. Ma J. (2020). Topical application of temperature-sensitive caerin 1.1 and 1.9 gel inhibits TC-1 tumor growth in mice. *Am. J. Transl. Res.* 12 191–202. 32051748 PMC7013226

[B39] McGregor S. Saulo D. Brotherton J. M. L. Liu B. Phillips S. Skinner S. R. (2018). Decline in prevalence of human papillomavirus infection following vaccination among Australian indigenous women, a population at higher risk of cervical cancer: the VIP-I study. *Vaccine* 36 4311–4316. 10.1016/j.vaccine.2018.05.104 29880245

[B40] Nakajima S. Fujiwara T. Ohguchi H. Onishi Y. Kamata M. Okitsu Y. (2014). Induction of thymic stromal lymphopoietin in mesenchymal stem cells by interaction with myeloma cells. *Leuk. Lymphom.* 55 2605–2613. 10.3109/10428194.2014.881478 24410591

[B41] Ni G. Liang D. Cummins S. F. Walton S. F. Chen S. Wang Y. (2018). Comparative proteomic study of the antiproliferative activity of frog host-defence peptide caerin 1.9 and its additive effect with caerin 1.1 on TC-1 cells transformed with HPV16 E6 and E7. *Biomed. Res. Int.* 2018:7382351. 10.1155/2018/7382351 PMC5971270 29862288

[B42] Ni G. Wang T. Walton S. Zhu B. Chen S. Wu X. (2015). Manipulating IL-10 signalling blockade for better immunotherapy. *Cell Immunol.* 293 126–129. 10.1016/j.cellimm.2014.12.012 25596475

[B43] Ni M. Chen Y. Lim E. Wimberly H. Bailey S. T. Imai Y. (2011). Targeting androgen receptor in estrogen receptor-negative breast cancer. *Cancer Cell* 20 119–131. 10.1016/j.ccr.2011.05.026 21741601 PMC3180861

[B44] Nishina H. Wada T. Katada T. (2004). Physiological roles of SAPK/JNK signaling pathway. *J. Biochem.* 136 123–126. 10.1093/jb/mvh117 15496581

[B45] Orhan G. Bayram A. Zer Y. Balci I. (2005). Synergy tests by E test and checkerboard methods of antimicrobial combinations against *Brucella melitensis*. *J. Clin. Microbiol.* 43 140–143. 10.1128/jcm.43.1.140-143.2005 15634962 PMC540140

[B46] Pan X. Ma B. You X. Chen S. Wu J. Wang T. (2019). Synthesized natural peptides from amphibian skin secretions increase the efficacy of a therapeutic vaccine by recruiting more T cells to the tumour site. *BMC Complem. Altern. Med.* 19:163. 10.1186/s12906-019-2571-z 31277636 PMC6612097

[B47] Pedroza-Gonzalez A. Xu K. Wu T. C. Aspord C. Tindle S. Marches F. (2011). Thymic stromal lymphopoietin fosters human breast tumor growth by promoting type 2 inflammation. *J. Exp. Med.* 208 479–490. 10.1084/jem.20102131 21339324 PMC3058586

[B48] Perez-Riverol Y. Csordas A. Bai J. Bernal-Llinares M. Hewapathirana S. Kundu D. J. (2019). The PRIDE database and related tools and resources in 2019: improving support for quantification data. *Nucleic Acids Res.* 47 D442–D450. 30395289 10.1093/nar/gky1106 PMC6323896

[B49] Petersen S. L. Wang L. Yalcin-Chin A. Li L. Peyton M. Minna J. (2007). Autocrine TNFalpha signaling renders human cancer cells susceptible to Smac-mimetic-induced apoptosis. *Cancer Cell* 12 445–456. 10.1016/j.ccr.2007.08.029 17996648 PMC3431210

[B50] Raulf N. Lucarelli P. Thavaraj S. Brown S. Vicencio J. M. Sauter T. (2018). Annexin A1 regulates EGFR activity and alters EGFR-containing tumour-derived exosomes in head and neck cancers. *Eur. J. Cancer* 102 52–68. 10.1016/j.ejca.2018.07.123 30142511

[B51] Shen J. Xiao Z. Zhao Q. Li M. Wu X. Zhang L. (2018). Anti-cancer therapy with TNFalpha and IFNgamma: a comprehensive review. *Cell Prolif.* 51:e12441. 10.1111/cpr.12441 29484738 PMC6528874

[B52] Sun H. Q. Yamamoto M. Mejillano M. Yin H. L. (1999). Gelsolin, a multifunctional actin regulatory protein. *J. Biol. Chem.* 274 33179–33182. 10.1074/jbc.274.47.33179 10559185

[B53] Sun S. Liang X. Zhang X. Liu T. Shi Q. Song Y. (2015). Phosphoglycerate kinase-1 is a predictor of poor survival and a novel prognostic biomarker of chemoresistance to paclitaxel treatment in breast cancer. *Br. J. Cancer* 112 1332–1339. 10.1038/bjc.2015.114 25867275 PMC4402453

[B54] Szklarczyk D. Franceschini A. Wyder S. Forslund K. Heller D. Huerta-Cepas J. (2015). STRING v10: protein-protein interaction networks, integrated over the tree of life. *Nucleic Acids Res.* 43 D447–D452. 25352553 10.1093/nar/gku1003 PMC4383874

[B55] VanCompernolle S. E. Taylor R. J. Oswald-Richter K. Jiang J. Youree B. E. Bowie J. H. (2005). Antimicrobial peptides from amphibian skin potently inhibit human immunodeficiency virus infection and transfer of virus from dendritic cells to T cells. *J. Virol.* 79 11598–11606. 10.1128/jvi.79.18.11598-11606.2005 16140737 PMC1212620

[B56] Vander Heiden M. G. Cantley L. C. Thompson C. B. (2009). Understanding the Warburg effect: the metabolic requirements of cell proliferation. *Science* 324 1029–1033. 10.1126/science.1160809 19460998 PMC2849637

[B57] Wang C. Yan R. Luo D. Watabe K. Liao D. F. Cao D. (2009). Aldo-keto reductase family 1 member B10 promotes cell survival by regulating lipid synthesis and eliminating carbonyls. *J. Biol. Chem.* 284 26742–26748. 10.1074/jbc.m109.022897 19643728 PMC2785362

[B58] Wang C. Y. Cusack J. C. Jr. Liu R. Baldwin A. S. Jr. (1999). Control of inducible chemoresistance: enhanced anti-tumor therapy through increased apoptosis by inhibition of NF-kappaB. *Nat. Med.* 5 412–417. 10.1038/7410 10202930

[B59] Wang J. Ying G. Wang J. Jung Y. Lu J. Zhu J. (2010). Characterization of phosphoglycerate kinase-1 expression of stromal cells derived from tumor microenvironment in prostate cancer progression. *Cancer Res.* 70 471–480. 10.1158/0008-5472.can-09-2863 20068185 PMC3086494

[B60] Wang L. Guo Y. Huang W. J. Ke X. Poyet J. L. Manji G. A. (2001). Card10 is a novel caspase recruitment domain/membrane-associated guanylate kinase family member that interacts with BCL10 and activates NF-kappa B. *J. Biol. Chem.* 276 21405–21409. 10.1074/jbc.m102488200 11259443

[B61] Wang X. Lu Y. Yang J. Shi Y. Lan M. Liu Z. (2008). Identification of triosephosphate isomerase as an anti-drug resistance agent in human gastric cancer cells using functional proteomic analysis. *J. Cancer Res. Clin. Oncol.* 134 995–1003. 10.1007/s00432-008-0367-5 18309519 PMC12160761

[B62] Wettstein G. Bellaye P. S. Micheau O. Bonniaud P. (2012). Small heat shock proteins and the cytoskeleton: an essential interplay for cell integrity? *Int. J. Biochem. Cell Biol.* 44 1680–1686. 10.1016/j.biocel.2012.05.024 22683760

[B63] Witton C. J. Reeves J. R. Going J. J. Cooke T. G. Bartlett J. M. (2003). Expression of the HER1-4 family of receptor tyrosine kinases in breast cancer. *J. Pathol.* 200 290–297. 10.1002/path.1370 12845624

[B64] Xie F. Meng Y. H. Liu L. B. Chang K. K. Li H. Li M. Q. (2013). Cervical carcinoma cells stimulate the angiogenesis through TSLP promoting growth and activation of vascular endothelial cells. *Am. J. Reprod. Immunol.* 70 69–79. 10.1111/aji.12104 23495958

[B65] Yan Y. B. (2016). Creatine kinase in cell cycle regulation and cancer. *Amino Acids* 48 1775–1784. 10.1007/s00726-016-2217-0 27020776

[B66] Zha X. Wang F. Wang Y. He S. Jing Y. Wu X. (2011). Lactate dehydrogenase B is critical for hyperactive mTOR-mediated tumorigenesis. *Cancer Res.* 71 13–18. 10.1158/0008-5472.can-10-1668 21199794

[B67] Zieker D. Konigsrainer I. Tritschler I. Loffler M. Beckert S. Traub F. (2010). Phosphoglycerate kinase 1 a promoting enzyme for peritoneal dissemination in gastric cancer. *Int. J. Cancer* 126 1513–1520. 19688824 10.1002/ijc.24835 PMC2811232

