# Supplementary figures and images for: Host-Defense Peptides Caerin 1.1 and 1.9 Stimulate TNF-Alpha-Dependent Apoptotic Signals in Human Cervical Cancer HeLa Cells
Source: Front Cell Dev Biol. 2020 Jul 31;8:676. doi: 10.3389/fcell.2020.00676 (PMC7412766; doi:10.3389/fcell.2020.00676)

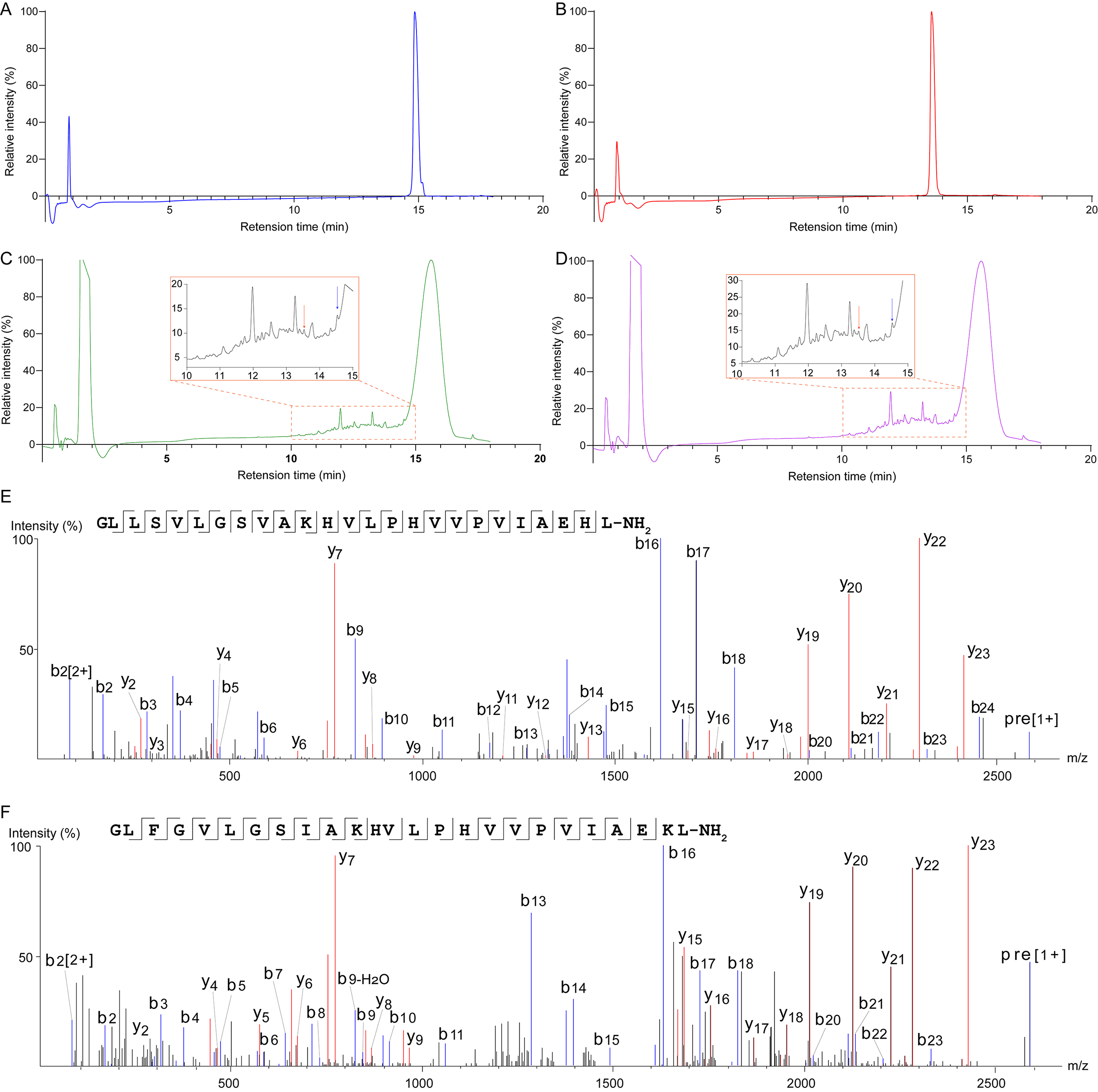

Supplement: FIGURE S1 — Identification of caerin 1.1 and 1.9 in the cytoplasm of HeLa cells. The chromatograms of synthetic caerin 1.1 (A), caerin 1.9 (B), cytoplasmic protein extracts (MW <10kDa) of HeLa cells treated by 3.9 nM caerin 1.1/1.9 (1:1 molar ration) at 1 hr (C) and 2 hr (D). Arrows indicate the peaks potentially corresponding to caerin 1.1 or 1.9, respectively. The selected MS/MS spectra of caerin 1.1 (E) and 1.9 (F) identified in the 30s fraction collected around the potential peaks in cytoplasm of HeLa cells. [file Image_1.TIF]

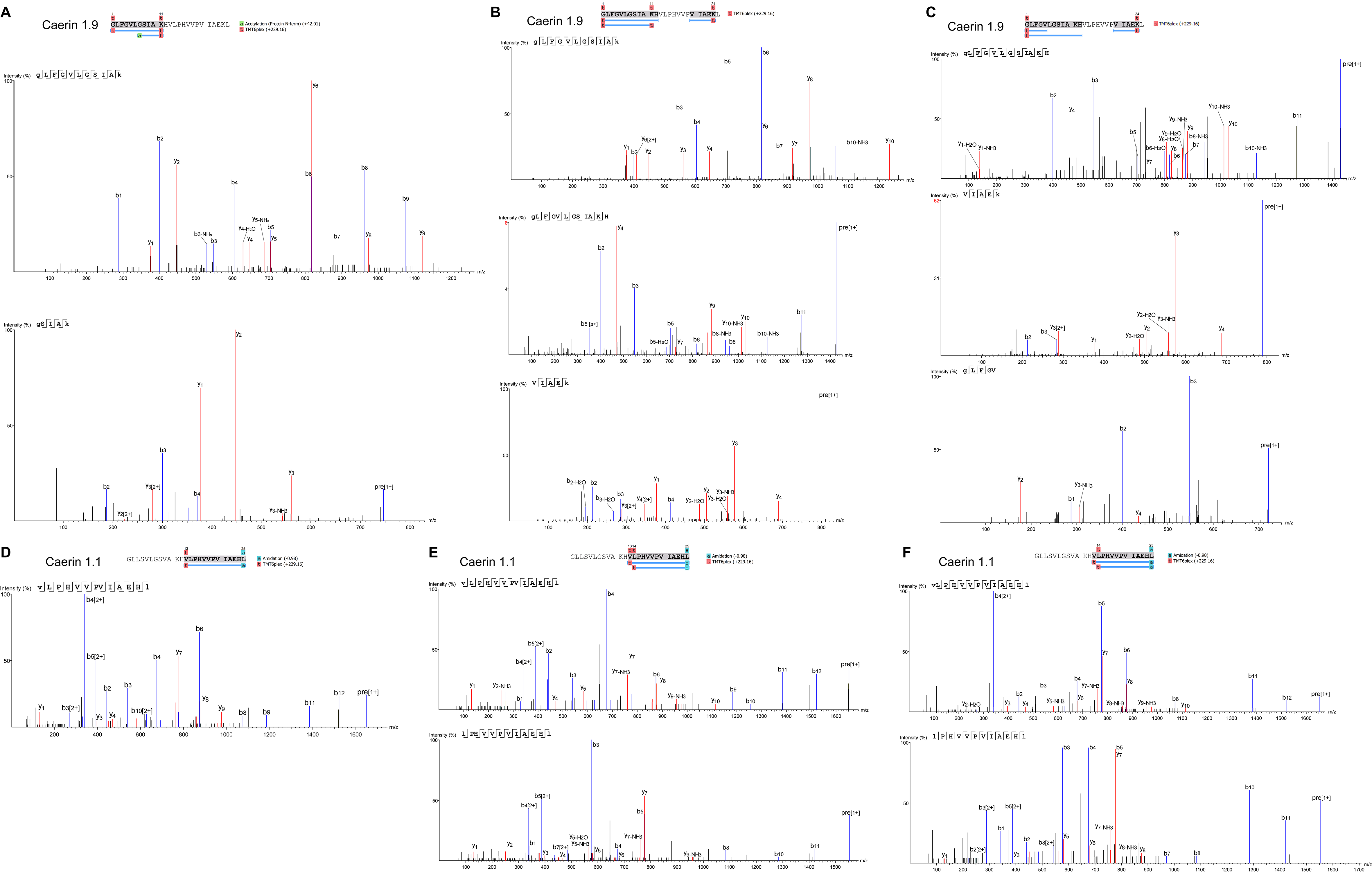

Supplement: FIGURE S2 — Identification of caerin 1.9 in HeLa cells and caerin 1.1 in cell environment post TMT labelling. The coverage of caerin 1.9 sequence and MS/MS spectra of supporting peptides in cell samples, (A) replicate 1, (B) replicate 2 and (C) replicate 3. The coverage of caerin 1.1 sequence and MS/MS spectra of supporting peptides in cell growth environment, (D) replicate 1, (E) replicate 2 and (F) replicate 3. [file Image_2.TIF]

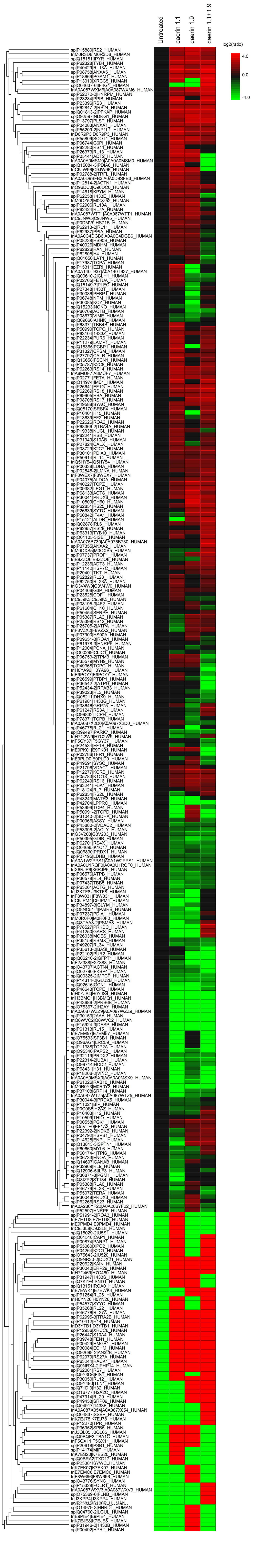

Supplement: FIGURE S3 — Hierarchy cluster of differentially expressed proteins with significance (fold change = 1.5) in HeLa cells, identified from TMT labelling analysis of biological replicate 1 treated with caerin 1.1, 1.9 and 1.1/1.9 with reference to untreated cells at 24 h. The magnitudes of up-/down- regulation (Log2FC values) are indicated by color change. The fold change data was listed in Supplementary Table 4. [file Image_3.TIF]

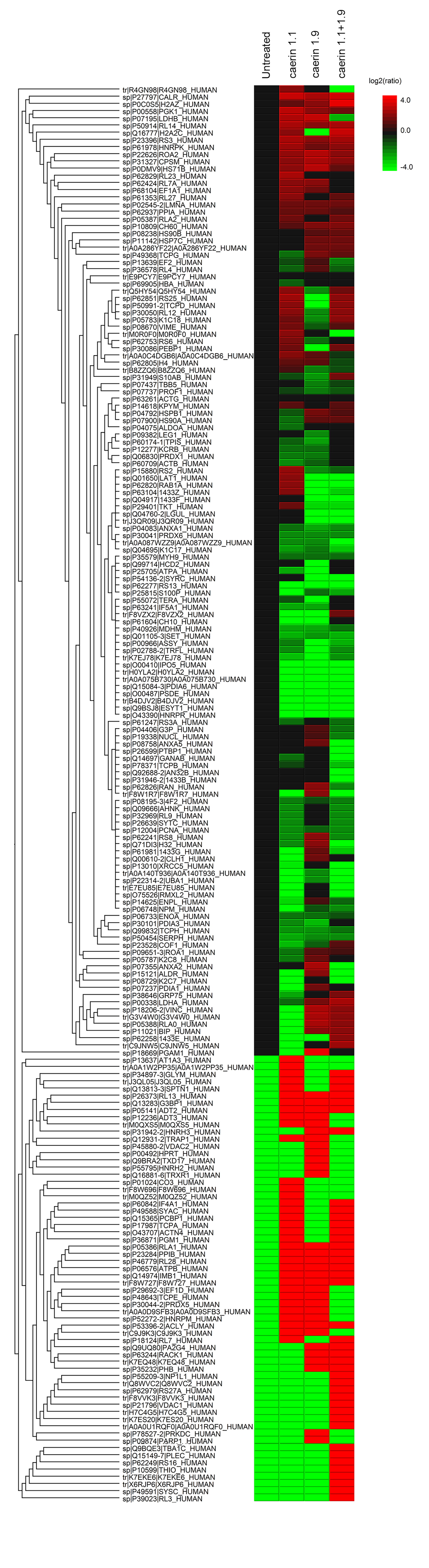

Supplement: FIGURE S4 — Hierarchy cluster of differentially expressed proteins with significance (fold change = 1.5) in HeLa cells, identified from TMT labelling analysis of biological replicate 2 treated with caerin 1.1, 1.9 and 1.1/1.9 with reference to untreated cells at 24 h. The magnitudes of up-/down- regulation (Log2FC values) are indicated by color change. The fold change data was listed in Supplementary Table 4. [file Image_4.TIF]

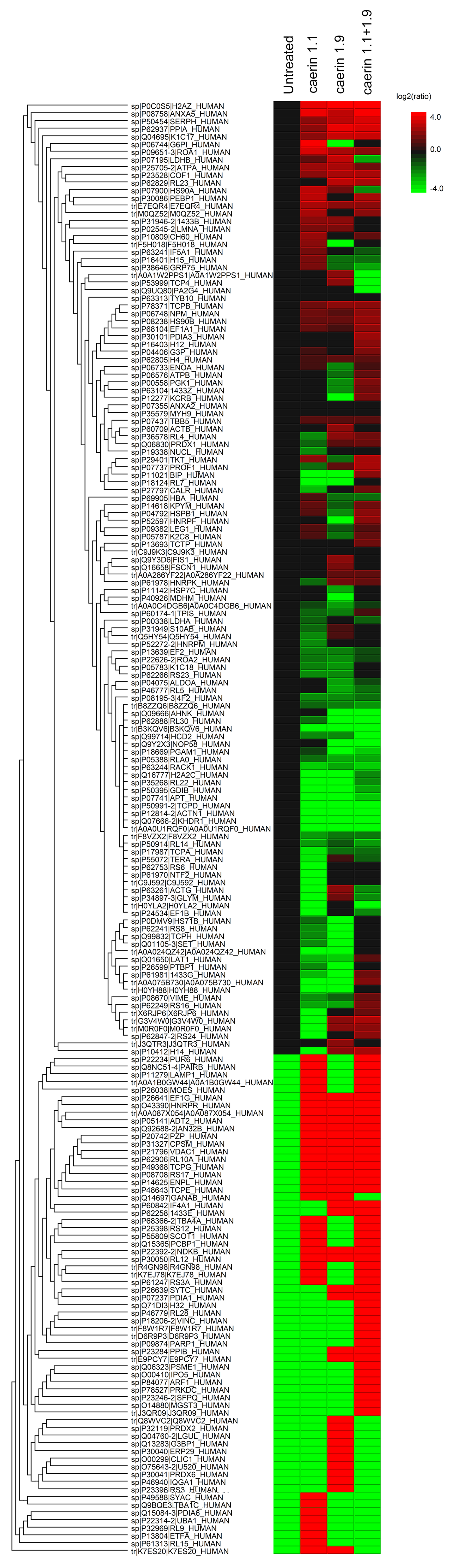

Supplement: FIGURE S5 — Hierarchy cluster of differentially expressed proteins with significance (fold change = 1.5) in HeLa cells, identified from TMT labelling analysis of biological replicate 3 treated with caerin 1.1, 1.9 and 1.1/1.9 with reference to untreated cells at 24 h. The magnitudes of up-/down- regulation (Log2FC values) are indicated by color change. The fold change data was listed in Supplementary Table 4. [file Image_5.TIF]

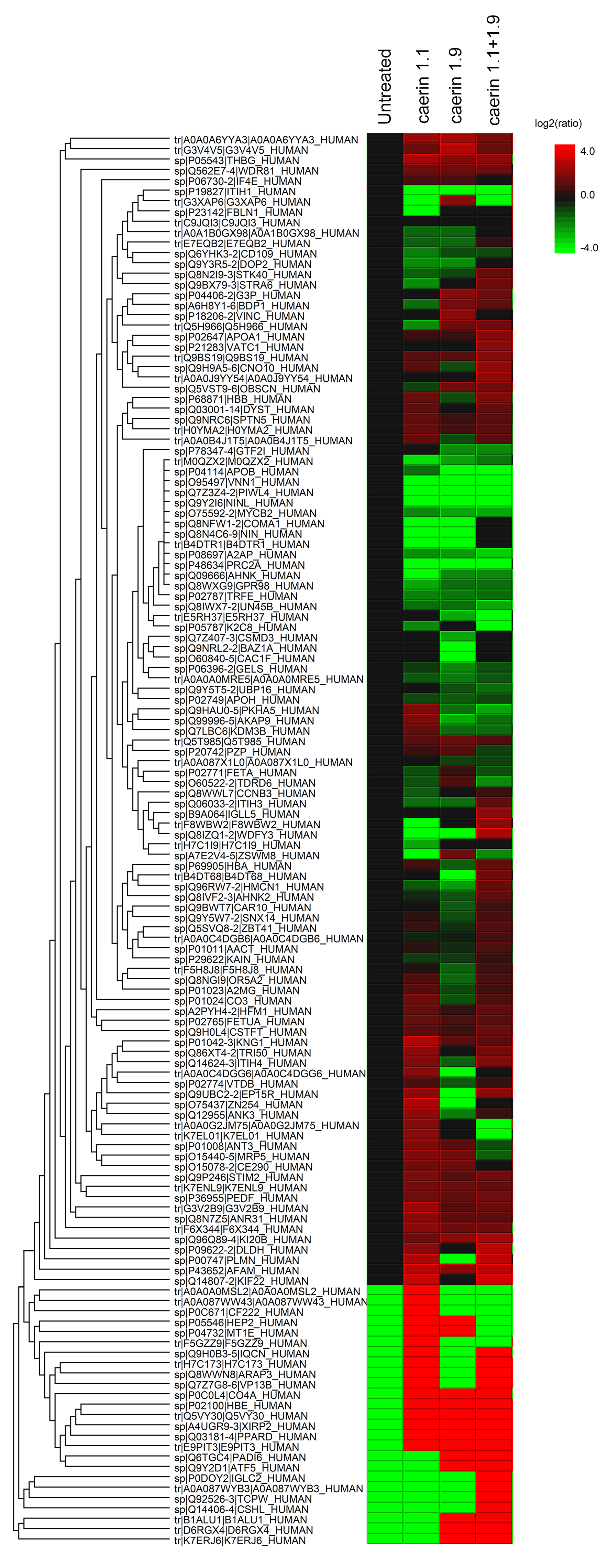

Supplement: FIGURE S6 — Hierarchy cluster of differentially expressed proteins with significance (fold change = 1.5) in the growth environment of HeLa cells, identified from TMT labelling analysis of biological replicate 1 treated with caerin 1.1, 1.9 and 1.1/1.9 with reference to those of untreated cells at 24 h. The magnitudes of up-/down- regulation (Log2FC values) are indicated by color change. The fold change data was listed in Supplementary Table 5. [file Image_6.TIF]

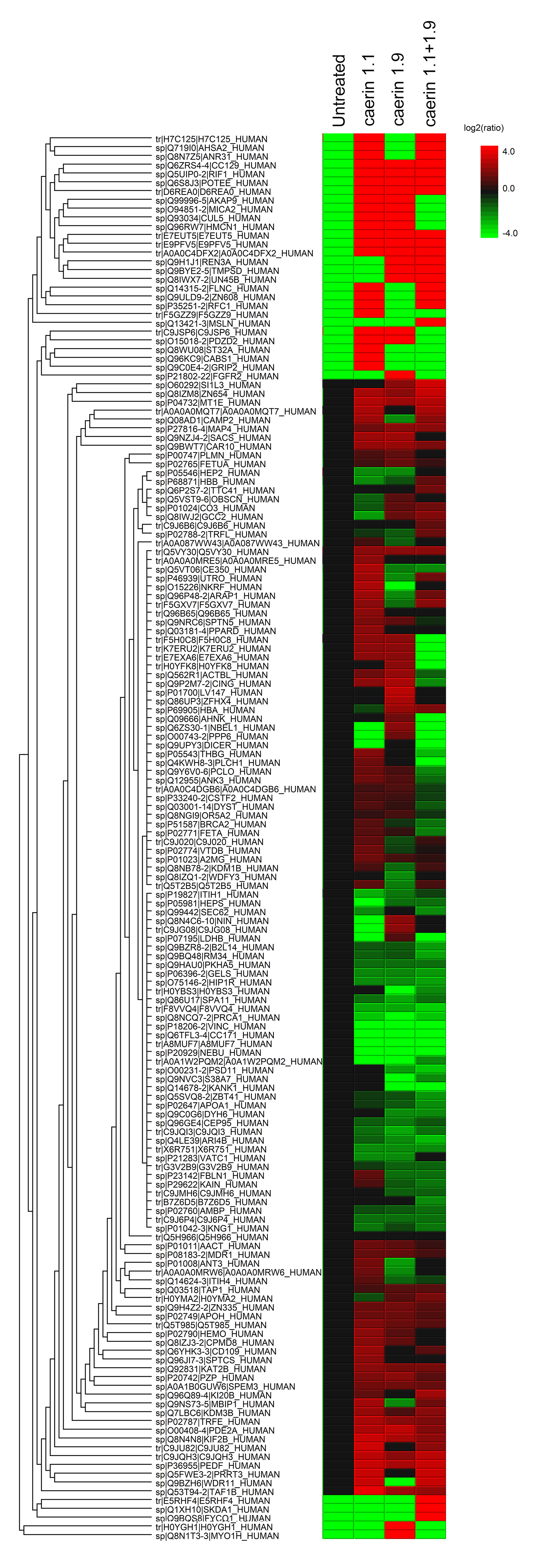

Supplement: FIGURE S7 — Hierarchy cluster of differentially expressed proteins with significance (fold change = 1.5) in the growth environment of HeLa cells, identified from TMT labelling analysis of biological replicate 2 treated with caerin 1.1, 1.9 and 1.1/1.9 with reference to those of untreated cells at 24 h. The magnitudes of up-/down- regulation (Log2FC values) are indicated by color change. The fold change data was listed in Supplementary Table 5. [file Image_7.TIF]

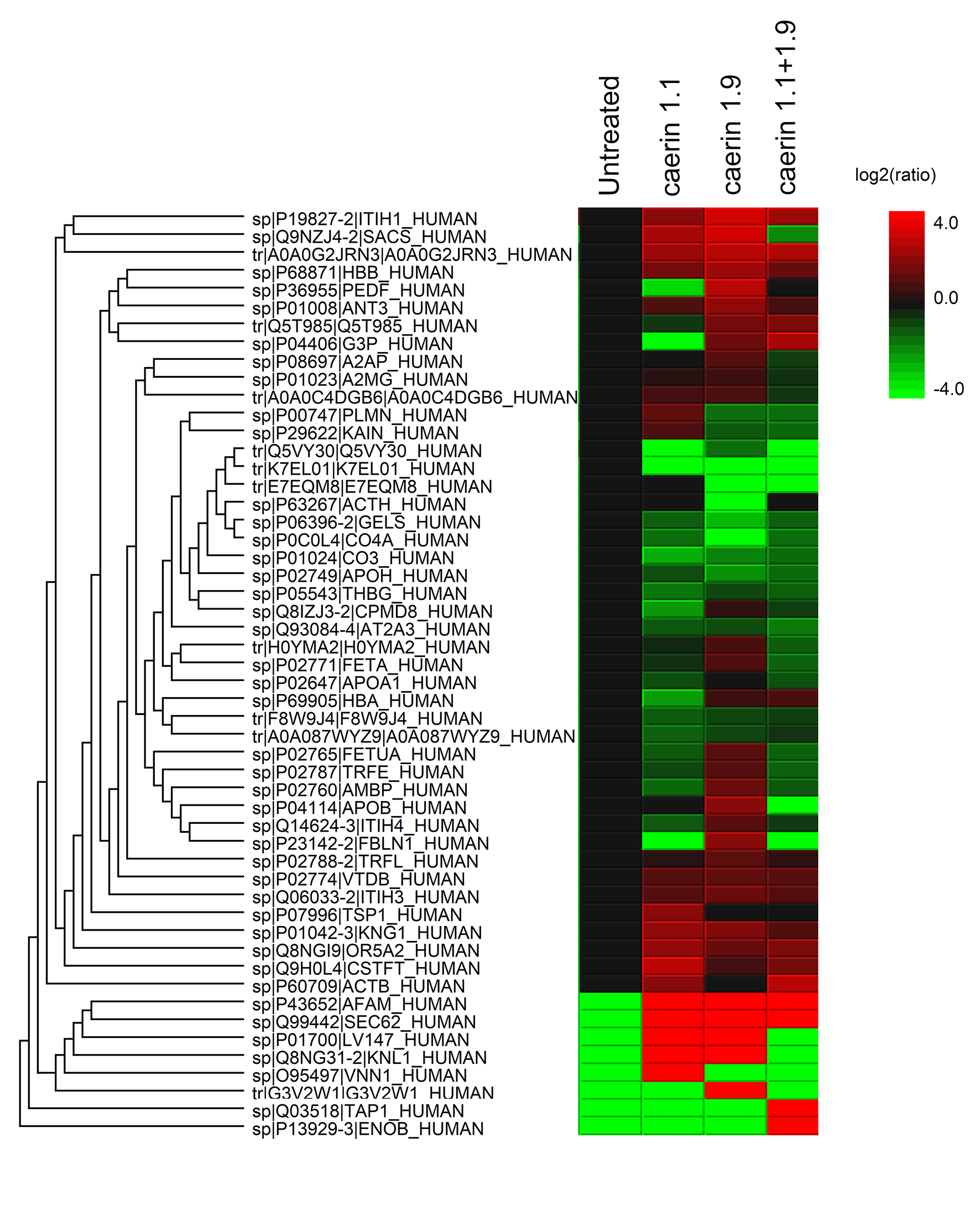

Supplement: FIGURE S8 — Hierarchy cluster of differentially expressed proteins with significance (fold change = 1.5) in the growth environment of HeLa cells, identified from TMT labelling analysis of biological replicate 3 treated with caerin 1.1, 1.9 and 1.1/1.9 with reference to those of untreated cells at 24 h. The magnitudes of up-/down- regulation (Log2FC values) are indicated by color change. The fold change data was listed in Supplementary Table 5. [file Image_8.TIF]

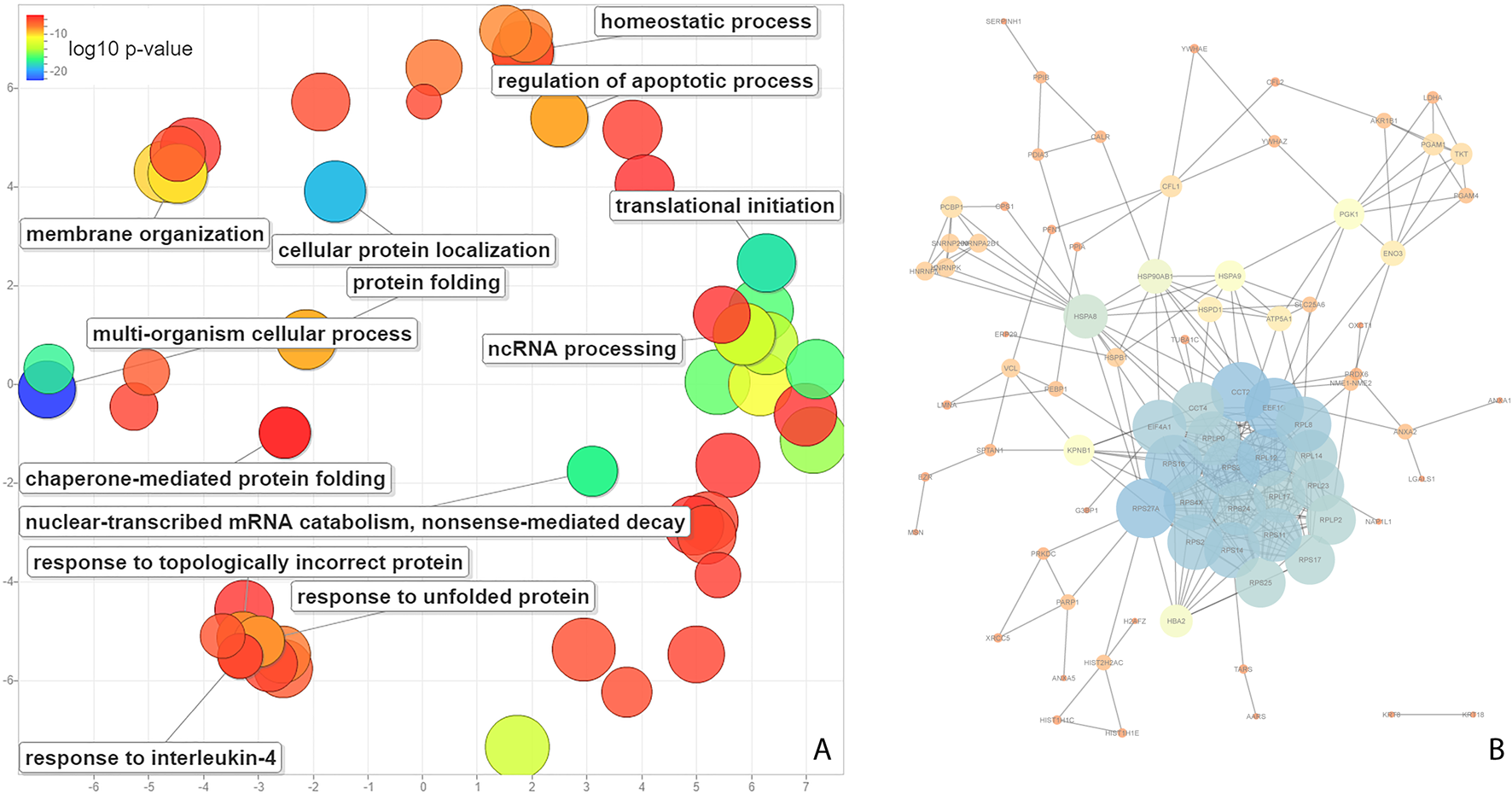

Supplement: FIGURE S9 — Enriched biological processes and PPIs of significantly upregulated proteins in cells treated with the mixture of caerin 1.1 and 1.9 (mass ratio 50:50). [file Image_9.TIF]

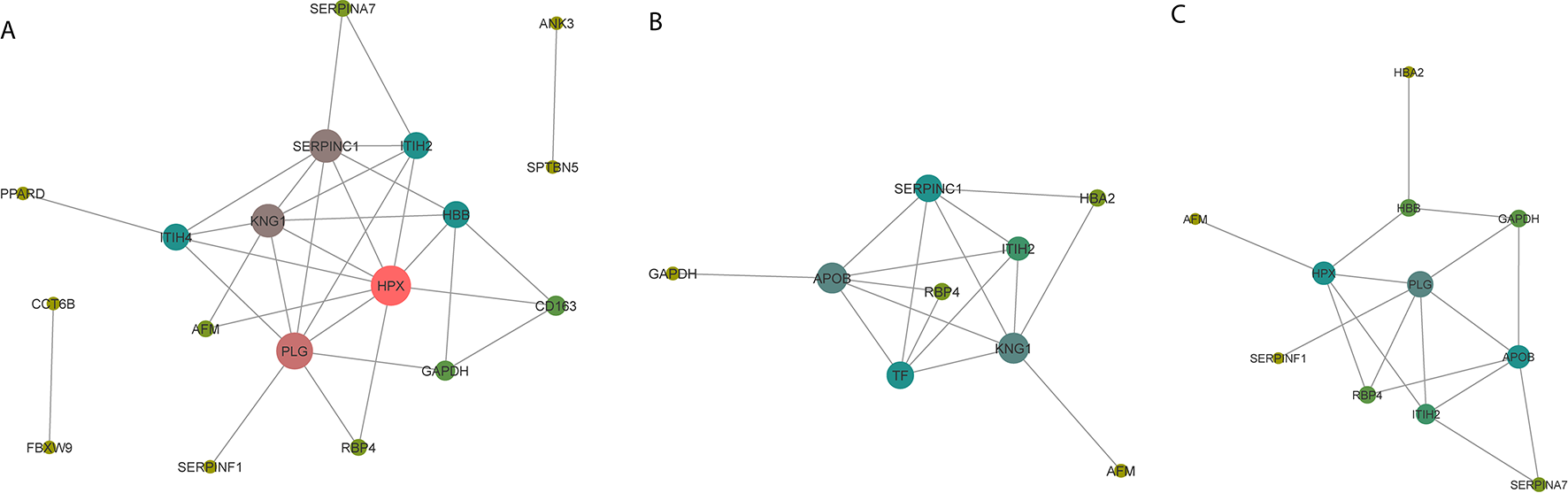

Supplement: FIGURE S10 — The enriched PPIs of proteins identified in the growth environment of HeLa cells with the peptide treatments. [file Image_10.TIF]

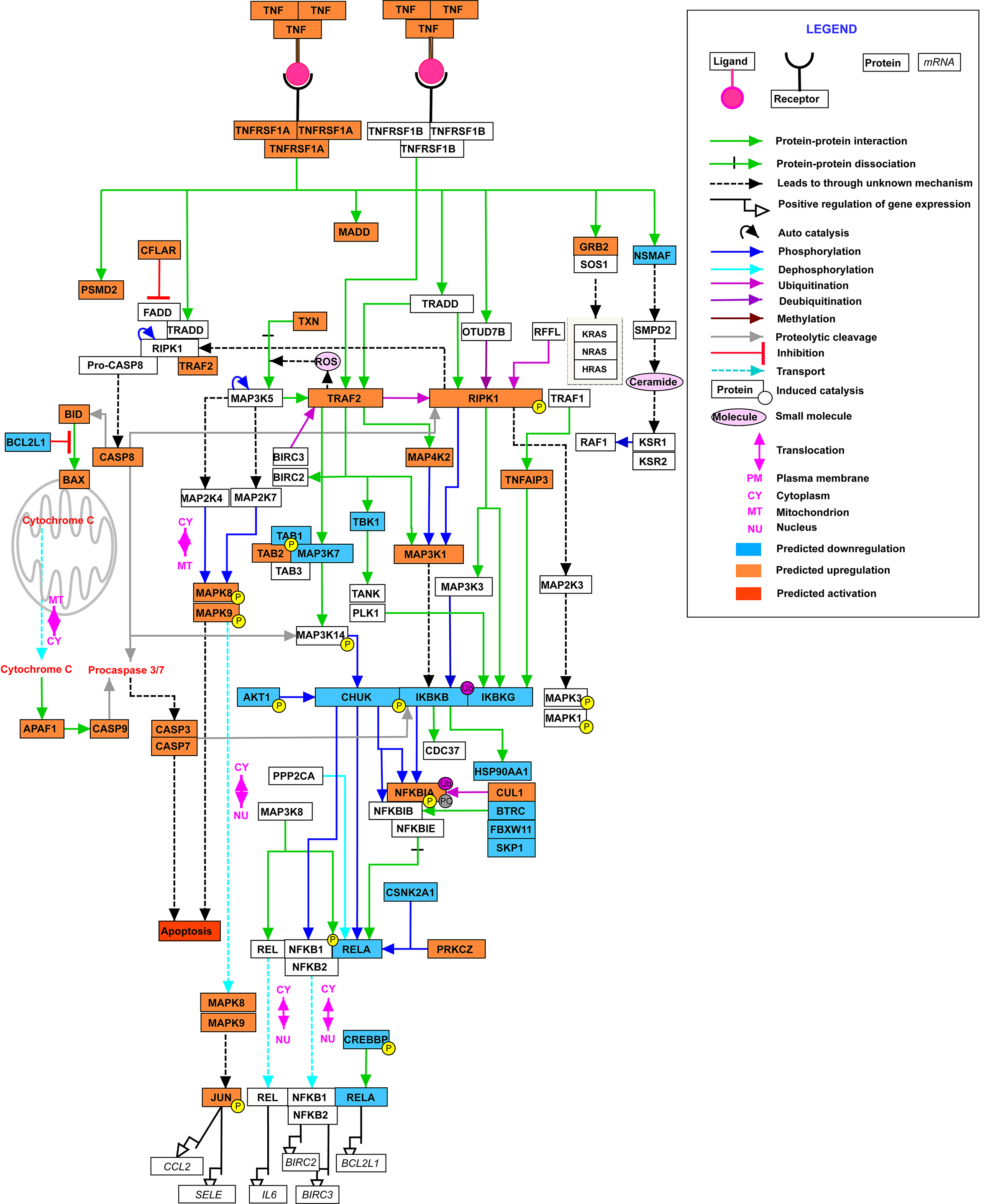

Supplement: FIGURE S11 — Caerin 1.1/1.9 treatment modulated TNFα signalling pathway. TNFα signalling pathway was identified as the canonical pathway in Hela cells significantly affected by the treatment of caerin 1.1/1.9, based on TMT-labelling data. Lines connecting the proteins represent known interactions, and arrows indicate the directions of up-/down-stream regulations. The pathway was downloaded and modified from WikiPathways (https://www.wikipathways.org/index.php/Pathway:WP231). [file Image_11.TIF]

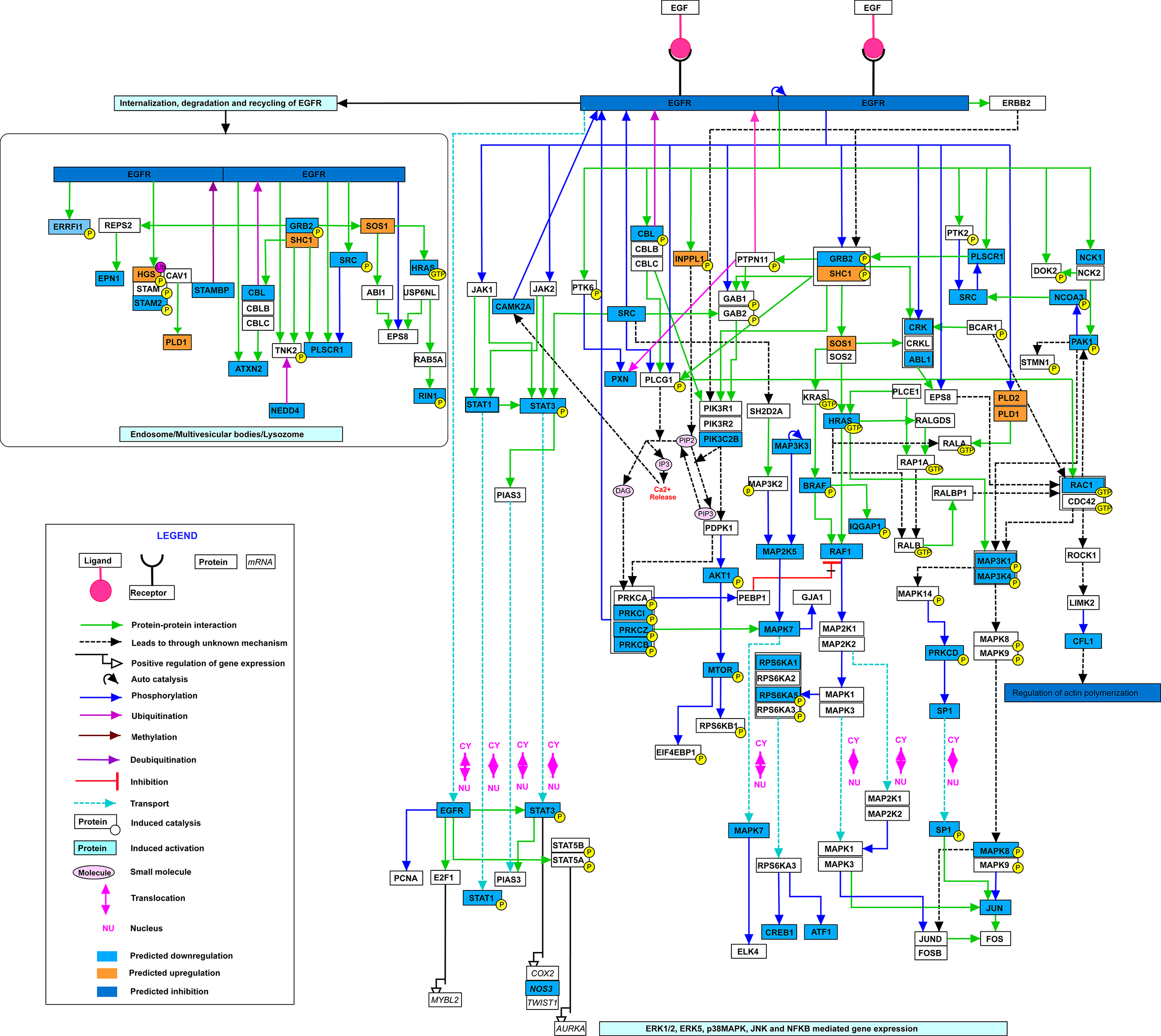

Supplement: FIGURE S12 — Caerin 1.1/1.9 treatment modulated EGFR signalling pathway. EGFR signalling pathway was identified as the canonical pathway in Hela cells significantly affected by the treatment of caerin 1.1/1.9, based on TMT-labelling data. Lines connecting the proteins represent known interactions, and arrows indicate the directions of up-/down-stream regulations. The pathway was downloaded and modified from WikiPathways (https://www.wikipathways.org/index.php/Pathway:WP437). [file Image_12.TIF]

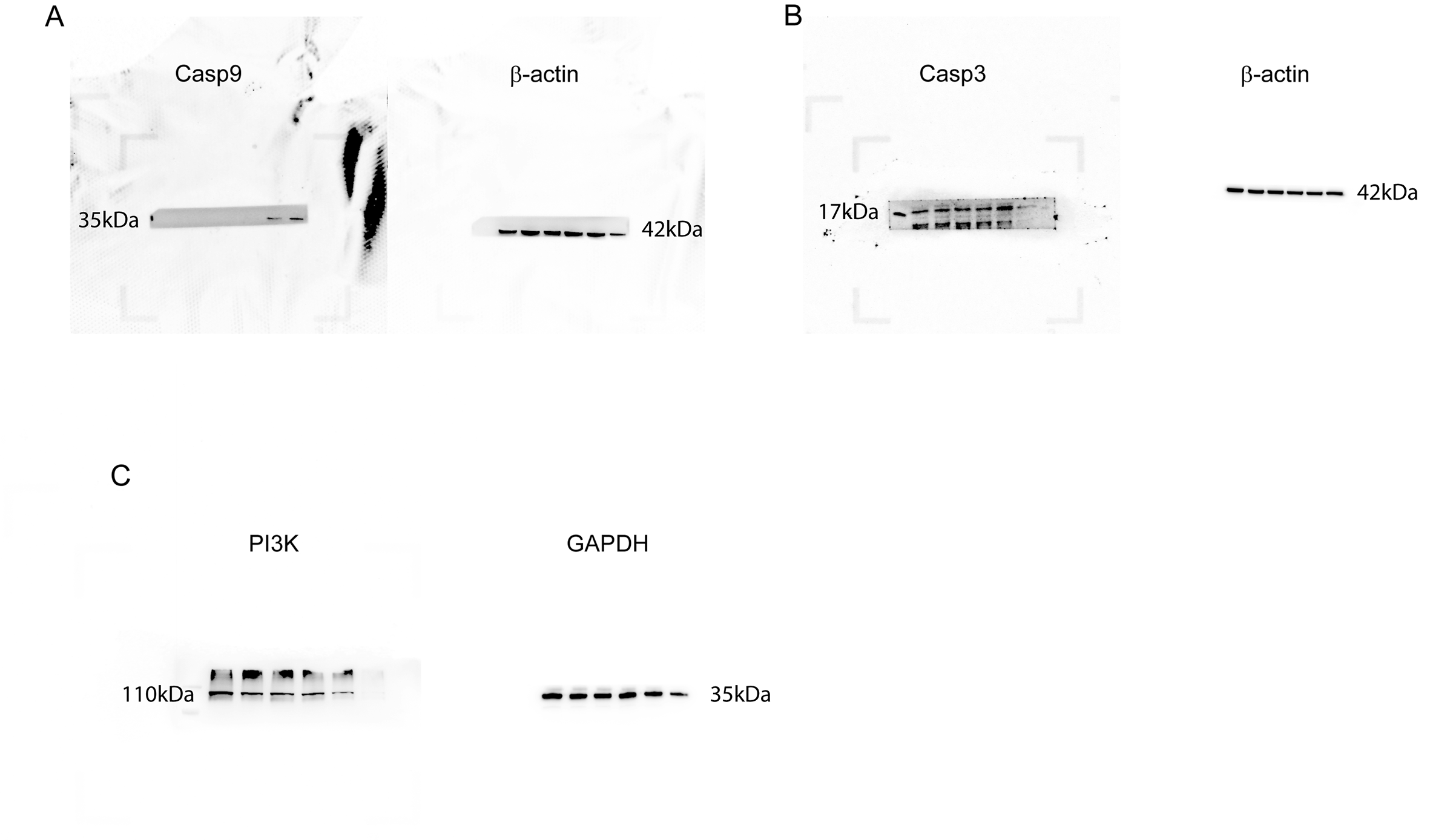

Supplement: FIGURE S13 — The original membrane images of western blot results shown in Figure 6, visualised by the image scanner ProteinSimple. From left to right, the blots corresponded to NC, 5, 10, 15, 20, and 25 μg/ml of peptide treatments, respectively. [file Image_13.TIF]
